# A Hypoxia Molecular Signature-Based Prognostic Model for Endometrial Cancer Patients

**DOI:** 10.3390/ijms24021675

**Published:** 2023-01-14

**Authors:** Yang Jiao, Rui Geng, Zihang Zhong, Senmiao Ni, Wen Liu, Zhiqiang He, Shilin Gan, Qinghao Huang, Jinhui Liu, Jianling Bai

**Affiliations:** 1 Department of Biostatistics, School of Public Heath, Nanjing Medical University, 101 Longmian Avenue, Jiangning District, Nanjing 211166, China; 2 Department of Gynecology, Nanjing Medical University, 101 Longmian Avenue, Jiangning District, Nanjing 211166, China

**Keywords:** endometrial cancer, hypoxia, tumor microenvironment (TME), prognosis, risk model, immune cells, chemotherapy, targeted treatment

## Abstract

Endometrial cancer has the highest incidence of uterine corpus cancer, the sixth most typical cancer in women until 2020. High recurrence rate and frequent adverse events were reported in either standard chemotherapy or combined therapy. Hence, developing precise diagnostic and prognostic approaches for endometrial cancer was on demand. Four hypoxia-related genes were screened for the EC prognostic model by the univariate, LASSO, and multivariate Cox regression analysis from the TCGA dataset. QT-PCR and functional annotation analysis were performed. Associations between predicted risk and immunotherapy and chemotherapy responses were investigated by evaluating expressions of immune checkpoint inhibitors, infiltrated immune cells, m6a regulators, and drug sensitivity. The ROC curve and calibration plot indicated a fair predictability of our prognostic nomogram model. NR3C1 amplification, along with IL-6 and SRPX suppressions, were detected in tumor. High stromal score and enriched infiltrated aDCs and B cells in the high-risk group supported the hypothesis of immune-deserted tumor. Hypoxia-related molecular subtypes of EC were then identified via the gene signature. Cluster 2 patients showed a significant sensitivity to Vinblastine. In summary, our hypoxia signature model accurately predicted the survival outcome of EC patients and assessed translational and transcriptional dysregulations to explore targets for precise medical treatment.

## 1. Introduction

Endometrial cancer (EC) has a high incidence rate among the subclassification of uterine corpus cancer until 2020 [1]. Unfortunately, early screening mainly focused on abnormal bleeding and might require additional evaluations like the pipelle method with high accuracy but sampling difficulty [2,3,4]. Patients diagnosed with stage III or IV endometrial cancer achieved a locoregional recurrence rate of 20% while treated with standard chemotherapy (doxorubicin-cisplatin (AP)) [5]. Additionally, In a randomized phase 3 trial, 58% of cases were reported with adverse events in chemo-radiotherapy and 63% of cases were reported in chemotherapy-only [6]. Hence, there is a growing need for developing endometrial cancer diagnostic and prognostic approaches.

While targeted therapies for endometrial cancer regarding glucose metabolism and the PI3K/Akt/mTOR pathway have been developed, there are rising concerns about a synchronous disturbance on other biological pathways of drugs [7,8]. Therefore, a high recurrence rate of endometrial cancer after radiotherapy and chemotherapy can be a result of tumor cell proliferation and angiogenesis [9]. According to previous studies, endometrial cancer was stratified into copy-number high, DNA-polymerase epsilon, microsatellite instability hyper mutated, and copy number low [10,11]. Moreover, the sequencing-based classification shows a potential association between molecular characteristics under hypoxia and adjuvant treatment for patients with high-grade tumors. 

The hypoxic tumor microenvironment always leads to poorer clinical results as tumor cells adapt to conditions of low oxygen and nutrition and become resistant to radiation and chemotherapy [12,13]. Recently, some studies indicated that hypoxia influences tumor cells in metabolism and immunity, thus resulting in immune infiltration and acidosis [14,15]. Therefore, novel molecular subtypes capable of distinguishing patients with similar histologic characteristics under hypoxic conditions is needed for customized treatments [16,17].

Specifically, targeting hypoxia in tumor cells gives rise to the development of immunotherapy via controlling immunosuppressive cells and effector T cells [18]. However, whether the genes related to the hypoxic tumor microenvironment can systematically contribute to the increased risk of endometrial cancer is unclear. 

Our study obtained the gene and clinical-relevant data of endometrial cancer patients from the Cancer Genome Atlas dataset. Several bioinformatic programs and packages were used in analyses, including linear models for microarray data (limma), clusterProfiler r packages and the Cell type identification by estimating relative subsets of RNA transcripts (CIBERSORT) algorithm. Limma has recently become famous for identifying differentially expressed mRNAs with thresholds of fold changes in an unsupervised clustering of samples [19,20]. In the annotation function analysis, the clusterProfiler package provides a comprehensive way to compare essential biological pathways among the classified gene set [21]. We established a hypoxia gene signature to calculate risk scores for patients and identified particular molecular subtypes of endometrial cancer from the samples. The nomogram consisted of risk scores, and several clinical characteristics were finally built for prognosis. To look into the potential benefits of existing therapies and the new targets of the treatment, we evaluated the immune cell infiltration by CIBERSORT algorithm, immune checkpoints by Estimation of stromal and immune cells in malignant tumors using expression data (ESTIMATE) algorithm, and the semi-inhibitory concentration (IC50) in drug sensitivity of chemotherapy. CIBERSORT has great power over computing infiltrating immune fractions with 22 immune cell types by the deconvolution of genetic microarray expression profiles and defining the immune phenotypes with signature genes from the TCGA samples. In Wang et al., the authors investigated the tumor-infiltrated immune cell levels and characteristics of tumor microenvironment for the constructed circRNA signature via CIBERSORT and ESTIMATE algorithms [22]. Practically, phenotype-genotype-dependent subtyping of EC provided an insight into the proper selection of suitable patients and their follow-ups into personalized therapies.

## 2. Results

### 2.1. Differential Expression Profile and Gene Enrichment Analysis of Hypoxia-Related DEGs in EC

**Figure 1:**
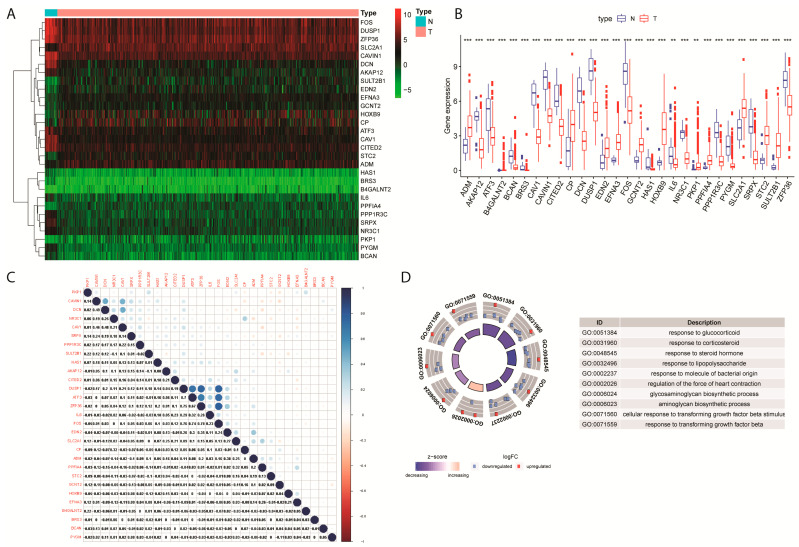
Differential expression profile of hypoxia-related genes in endometrial carcinoma. (**A**) heatmap of differentially expressed hypoxia-related genes clustered in N (Normal) and T (Tumor) cell types. (**B**) Differential expression of hypoxia related genes between N (Normal) and T (Tumor) cell types. ** means *p* < 0.01, *** means *p* < 0.001. (**C**) Correlation matrix plot of hypoxia-related differential expressed genes. (**D**) Gene Ontology (GO) Functional Annotation analysis of 29 hypoxia-related DEGs.

In the TCGA-EC cohort, 29 hypoxia-related differentially expressed genes (DEGs) with FDR < 0.001 were retained for further analysis (Figure 1A, Table S1), including 12 genes up-regulated and 17 down-regulated (Figure S1A). As explicitly shown in the heatmap, differential expression profiles of the 29 DEGs related to hypoxia were exhibited in normal or endometrial tumor cell types (Figure 1B). Several hypoxia-related DEGs showed a highly correlated relationship to their expression levels in tumor samples, such as FOS and DUSP-1 (Figure 1C). 

Function analysis results indicated that these DEGs could have immune-related roles (Figure 1D). From Figure S1B, one of the most significant functions was the response to steroid hormone, which involves seven DEGs. KEGG enrichment analysis results found that one out of two EC-related signaling pathways were significantly enriched by these DEGs (Figure S1C, Insulin resistance *p*-value = 0.008, Human T-cell leukemia virus 1 infection *p* = 0.061). 

### 2.2. Construction of a Prognostic Four-Gene Model for EC

**Figure 2:**
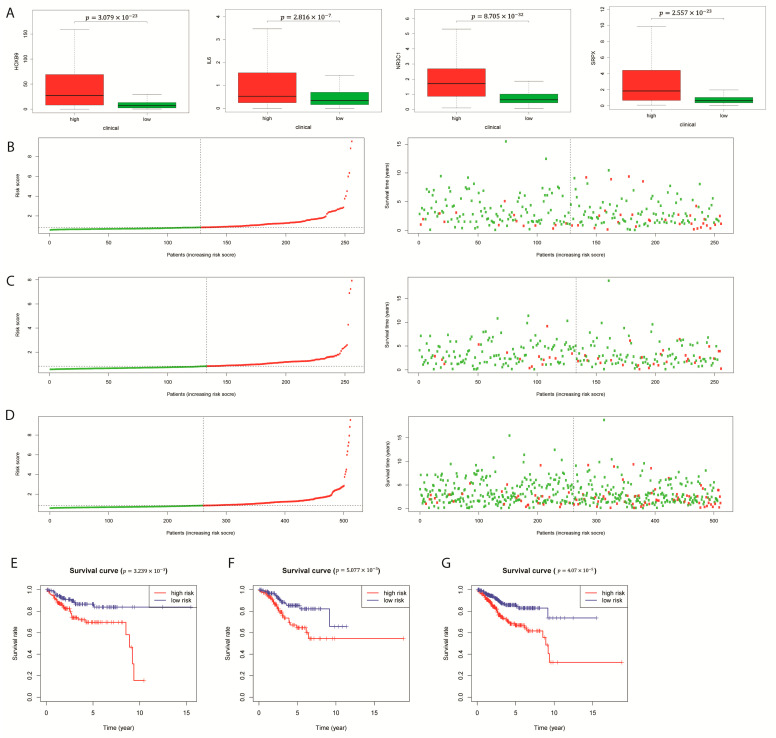
Development of prognostic model combining hypoxia-related gene signature and clinical factors. (**A**) mRNA Expression of gene HOXB9, IL6, NR3C1, and SRPX compared between low and high-risk groups. (**B**–**D**) The risk score rank (**left**) and distribution of survival status (**right**) of the four genes in the training set, testing set, and entire set. Green for alive and red for dead in high-risk groups. (**E**–**G**) Kaplan-Meier OS for high-risk group and low-risk group in the training set, testing set, and entire set (**from left to right**).

Through the Univariate Cox regression and least absolute shrinkage and selection operation (LASSO) analysis, four out of twenty-nine prognostic significant DEGs were obtained for constructing the hypoxia gene signature of EC (Figure S2). From the TCGA-EC cohort, 256 samples were defined as the training cohort, and 256 samples were for model testing. The four-gene signature prognostic model was constructed via Multivariate Cox regression analysis: Risk score = (0.062 × expression level of SRPX) + (0.016 × expression level of IL6) + (0.006 × expression level of HOXB9) + (0.155 × expression level of NR3C1) (Table S2). Based on the calculated risk scores, samples were divided into high- or low-risk groups. Principal components analysis (PCA) analysis for the testing and entire sets illustrated a fit of the model (Figure S3A. center, right). From the boxplots, four genes were all expressed differentially in two risk groups (Figure 2A, *p* < 0.001).

Patients with increasing risk scores had an observed possibility of death status (Figure 2B–D). Moreover, the K-M survival curves of the training, testing and entire sets implicated the high-risk group’s lower OS rate (Figure 2E–G, *p*-value < 0.001). To note, a drop was found between the 9th and 10th year of the high risk group, which may due to the randomized division of samples or a batch effect while TCGA collecting patients’ survival information. We also computed the survival probability of subdivisions in clinical factors in two risk groups and uncovered distinct patterns within the subdivisions of each clinical factor (Figure S3B).

The risk score estimated from the four-gene signature was then incorporated with the clinical characteristics for the further multivariate cox regression analysis. Moreover, the univariate and multivariate Cox model of training dataset corroborated the adequate predictability of the model with an independent variable, the stage factor (Table 1, *p* < 0.001). This result could suggest a complex relationship among patients’ age, histological type, grade, and hypoxia-related risk scores in EC. Consequently, the trained model was validated by the entire set, which included three factors: Stage, grade, and risk score (*p* < 0.05). 

In the receiver operating characteristic curves (ROC) analysis, the one-, three-, and five-year AUC were shown in Figure S3C. It is recommended to use the risk score model for facilitating molecular subtype-based diagnosis.

### 2.3. Evaluations of Immune Cells and Highlighted mRNA Modifications between Risk Groups of EC 

**Figure 3:**
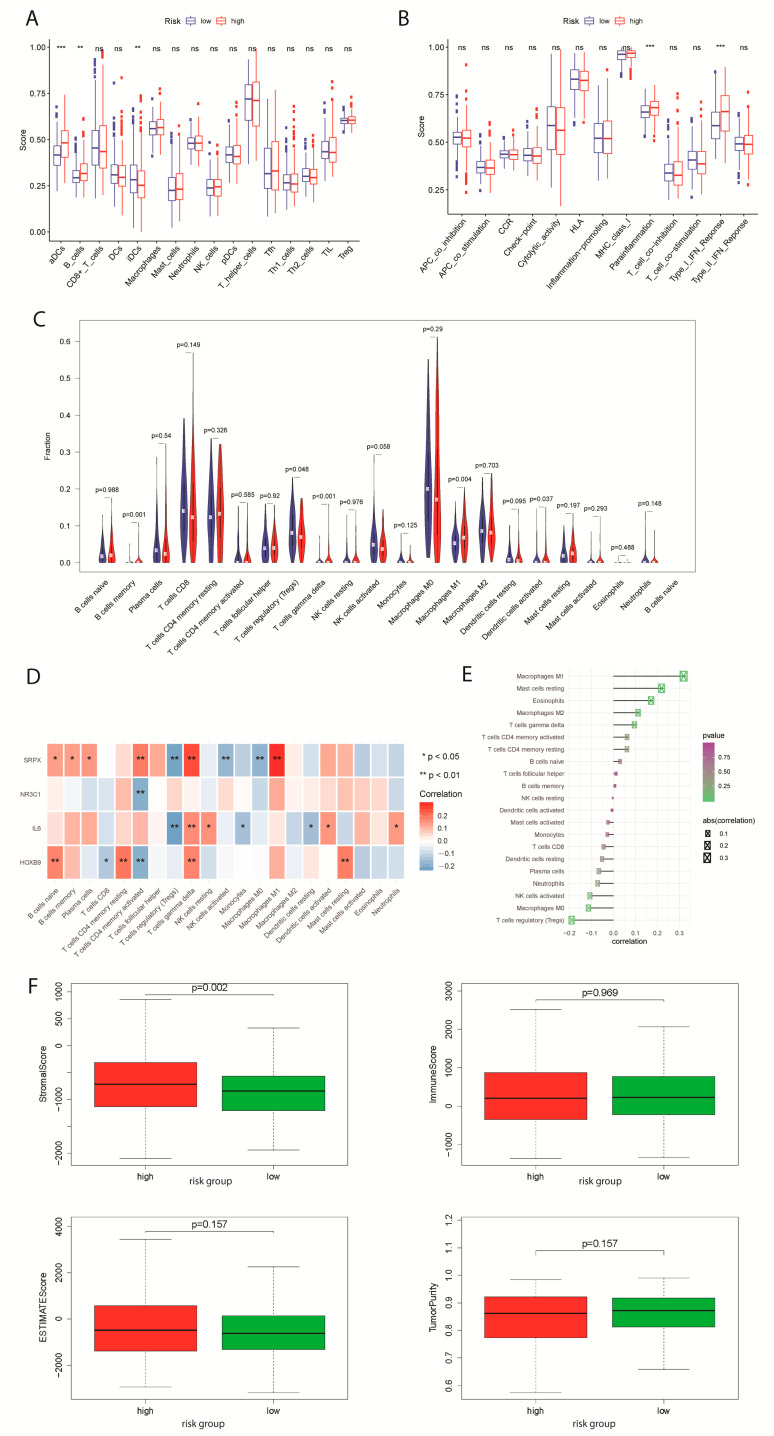
Infiltrating immune cells and tumor cell mRNA modifications correlated with hypoxia-related DEGs in EC patients. (**A**) The function scores of twenty-two immune cell types in EC samples between two risk groups. ** means *p* < 0.01; *** means *p* < 0.001. (**B**) The differences in thirteen immune pathways between two risk groups. (**C**) Abundance of 22 infiltrating immune cell types between two risk groups. (**D**) Correlation plot of 22 infiltrating immune cell types with four hypoxia-related DEGs in TCGA-EC cohort. (**E**) Correlation plot of 22 infiltrating immune cell types with four genes from the prognostic signature in TCGA-EC cohort. (**F**) Estimated Patterns of stromal cell scores (**up-left**), immune cell scores (**up-right**), ESTIMATE scores (**down-left**), and tumor purity (**down-right**) based on risk scores.

In order to study the related immune cells or pathways in EC, gene set variation analysis (GSVA) was performed to calculate the enrichment scores of low and high-risk groups of the EC patients in the TCGA cohort. In the gene enrichment of EC high-risk groups, human-activated dendritic cells (aDCs) and B cells were significantly differentiated among all measured immune cells (up-regulated) (Figure 3A). In contrast, human immature dendritic cells (iDCs) were highly upregulated in tissues of low-risk patients. Two immune pathways achieved elevated enrichment scores in EC high-risk groups: Parainflammation and Type I IFN response (Figure 3B). Recent studies showed that B cells could be a good indicator for the prolonged survival of high-grade EC patients. Besides, IgA regulation mediated by plgR in the EC tumor cells enhances the activation of inflammatory pathways involving IFN signaling and the hindrance to DNA repairing [23].

By the ESTIMATE algorithm, 22 types of immune cells were evaluated and eight were found significantly associated with the hypoxia gene signature (Figure S4A, *p* < 0.05). Specifically, gamma delta T cells and memory B cells were upregulated in the high-risk group with a high correlation (Figure 3C: *p* < 0.001, *p* = 0.001, Figure 3D: *p* = 0.00028, *p* = 0.0012), and Macrophages M1 was upregulated in the high-risk group with a correlation of *p* < 0.005 (Figure 3C:$\;p=3.47\times 10^{-5}$). Gene SRPX was highly expressed in the T cells gamma delta and Macrophages M1, which was consistent with the result of the GSVA (Figure 3D). Then, we calculated the correlation between the DEG expression profile in the signature and the immune cells in TCGA (Figure 3E). 

The high-risk group in EC has a moderately higher occupancy of stromal cells (Figure 3F(up-left), *p* < 0.005). However, neither high nor low-risk group showed significant differences between the immune cell fractions (Figure 3F(up-right)). Moreover, the undifferentiated estimate score and estimated tumor purity can be explained by the genetic heterogeneity of endometroid type or serous tumor (Figure 3F(down)). Besides, the subtype-specific immune cell expression clustered in levels of stromal, immune, and estimate scores, plus tumor purity, was presented (Figure S4B). To depict stemness featured in risk groups in EC samples, mRNA expression-based stemness index (mRNAsi) and epigenetically regulated mRNAsi (EREG-mRNAsi) were evaluated, and no significant differences were shown (Figure S4C). It suggested that the risk progression of the tumor was neither characterized by undifferentiating expression of cells nor co-expression regulations related to immune invasion but rather by simple stromal invasions resulting in the ectopic endometrial-like epithelium and stroma [24]. As a result, molecular mechanisms of stromal invasion in tumor tissues during pathogenesis are worth investigation for EC patients.

We later examine the expression level of the N6-methyladenosine (m6a) regulators compared between two risk groups. There were five m6a regulators significantly expressed in tumor tissues of high-risk patients, indicating possible epigenetic modifications or transcriptional dysregulations during EC tumorigenesis (Figure S4D).

### 2.4. Assessment of Tumor Microenvironment in Different Risk Groups of EC Samples

**Figure 4:**
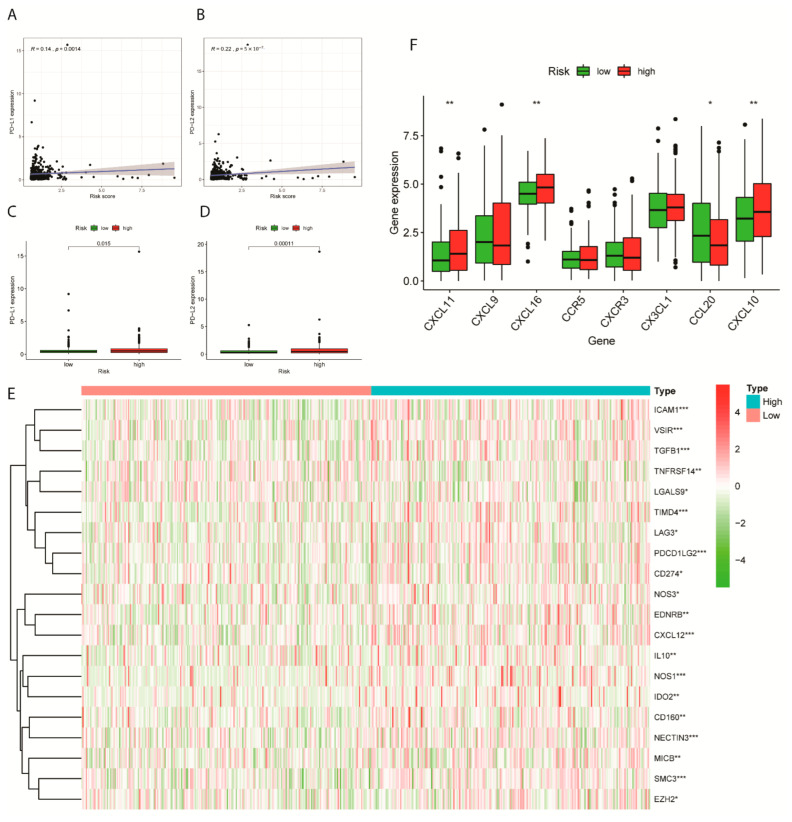
Immune checkpoint and immunosuppressive cytokine gene expression levels in high- and low-risk groups. (**A**) Scattered plot illustrating a correlation between the expression level of Immune checkpoint PD-L1 and risk scores in TCGA cohort. (**B**) Scattered plot illustrating a correlation between the expression level of Immune checkpoint PD-L2 and risk scores in TCGA cohort. (**C**) Expression level of PD-L1 compared between low and high-risk groups. (**D**) Expression level of PD-L2 compared between low and high-risk groups. (**E**) Heatmap of distinct immune checkpoint inhibitor marker expressed between low and high-risk groups. (**F**) Differential gene expression of the immunosuppressive cytokines in two risk groups from the TCGA-EC samples. * means *p* < 0.05; ** means *p* < 0.01; *** means *p* < 0.001.

Among 17 immune checkpoints, IDO1, ICOS, PD-L2, B7-H3, CD40, LAG3, CD86, PD-L1, and CD270 were differentially expressed in the low and high-risk groups of EC samples (Table 2, *p* < 0.05; Figure S5). Since PD-L1 and PD-L2 were reported to be promising candidates for immunotherapy [25,26], we therefore investigated expression profiles of immune checkpoint inhibitors (ICIs), PD-L1 and PD-L2 on patients in high and low risk-group from the TCGA cohort. The expression of PD-L1 and PD-L2 were positively correlated with patients’ risk scores with *p* < 0.005 (Figure 4A,B). Additionally, as indicated in the boxplots, the high-risk group achieved a relatively predominant expression of PD-L1 and PD-L2 (Figure 4C,D).

The following heatmap illustrating the risk-specific expression of immune checkpoint suppressors showed the complex modification of immuno-pathways, which can contribute to the pathological process and tumorigenesis (Figure 4E).

Cytokines functioned in inflammatory pathways and were reported to actively participate in EC pathogenesis. The high-risk group of EC samples disclosed substantial enrichment of CXCL11, CXCL16 and CCL20, while CXCL10 were upregulated in the low-risk group (Figure 4F).

### 2.5. TMB Evaluation and Chemotherapeutic Sensitivity Analysis in Prognostic Risk Groups 

**Figure 5:**
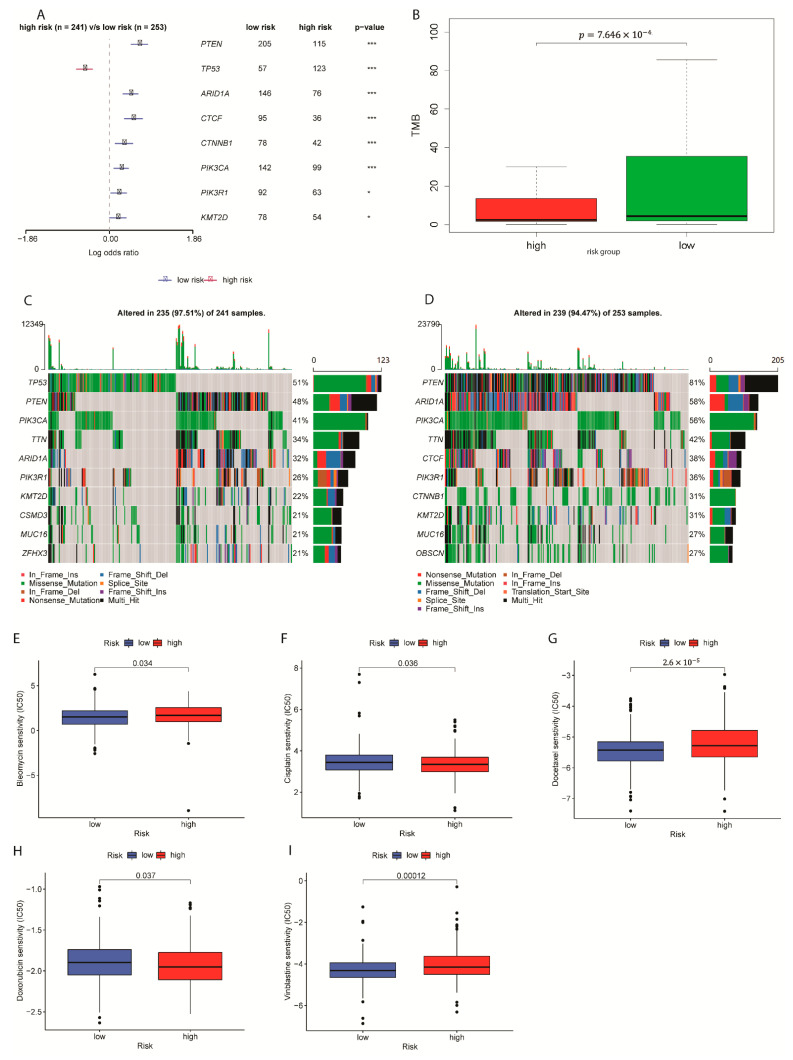
Gene mutation and drug sensitivity analysis in EC prognostic risk groups (**A**) Differential Expression of Top eight altered genes in risk groups. * means *p* < 0.05; *** means *p* < 0.001. (**B**) TMB for low and high-risk groups in the TCGA cohort. (**C**) Mutation profile of the high-risk group. (**D**) Mutation profile of the low-risk group. (**E**–**I**) Box plot of the estimated IC50 values for Bleomycin, Cisplatin, Docetaxel, Doxorubicin, and Vinblastine with two risk groups.

In the TCGA-EC cohort, 241 samples were sorted as high-risk, whereas 253 samples were as low-risk (Figure 5A). Eight genes were found for highly differential mutation frequencies between high and low-risk groups (*p* > 0.05). Tumor mutation burden (TMB) is significantly higher in the low-risk group indicating better prognostic immunotherapy benefits for EC cases in the low-risk group (Figure 5B). The correlation line with a *p*-value of 0.08999 (Figure S6, correlation index = −0.076). Thus, the prognostic model using only the risk score is recommended as a diagnostic tool and it is worthwhile to consider incorporating the clinical factors into the model.

Specifically, 97.51% of samples in the high-risk group had gene alterations, including missense mutation, such as mutations at gene TP53, while 94.47% of low-risk samples had gene alterations (Figure 5C,D and Figure S7).

Previous reports discovered influenced sensitivity of drugs Bleomycin, cisplatin, doxorubicin, and doxorubicin when either in a hypoxic condition or acidic conditions associated with hypoxia [27,28,29]. After patients’ responses to the chemotherapy were tested in terms of hypoxia-related genes signature, Bleomycin, Docetaxel, and Vinblastine displayed relatively higher sensitivity in high-risk EC samples (Figure 5E,G,I, *p* < 0.05). Treatments with Cisplatin and Doxorubicin displayed a lower sensitivity in high-risk samples (Figure 5F,H, *p* < 0.05). According to an article by Deschoemaeker et al., cisplatin resistance can be inverted with the removal of acidic stress, which resembles reoxygenation [30]. However, when compared with a normoxic condition, EC cells under hypoxic condition showed reduced sensitivity [31]. Therefore, while the majority of the chemotherapy results were validated, other sensitivity results need more studies related to hypoxic conditions to confirm in the endometrial cancer cases. 

### 2.6. Definition of Hypoxia Molecular Subtypes in EC for Diagnosis

**Figure 6:**
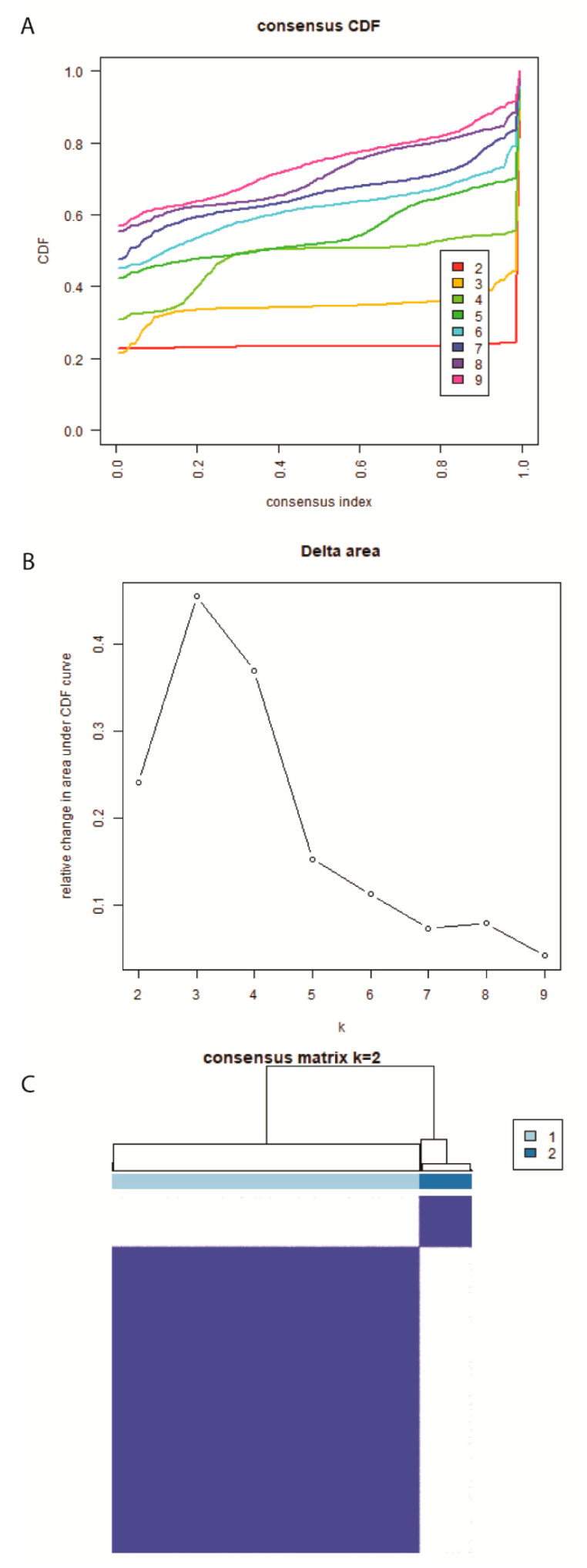
Consensus clustering of molecular subgroups in EC based on hypoxia-related DEGs (**A**) Cumulative distribution function (CDF) curve from k = 2 to k = 9. (**B**) CDF Delta area curve. The horizontal axis represents the number k and the vertical axis represents the relative change in the area under the CDF curve. (**C**) Consensus matrix heatmap of two clusters (k = 2) and the correlation area.

Accounting for the expression levels of four hypoxia-differentiated DEGs, EC samples were grouped from TCGA via The ConsensusClusterPlus package in R software. The consistent cumulative distribution function (CDF) graph and the delta region graph decided the optimal value of k, which is the cluster number (Figure 6A,B). When starting from k = 2, the consensus CDF curve is stable enough, corresponding to the insignificant delta area changes. Figure S8A explicitly illustrated the samples in TCGA allocated into CLUSTER 1 and CLUSTER 2 subtypes of EC and other possible clustering. Therefore, the heatmap in Figure 6C exhibited the consensus matrix when k = 2 in simplicity. The PCA and t-distributed stochastic neighbor embedding (t-SNE) results also agreed with our clustering outcome of two subtypes (Figure S8B,C). Further analysis of the tumor microenvironment and targets of adjuvant therapies featured with the hypoxia-related molecular subtypes will serve as suggestions for diagnosis use under the complex hypoxic TME. The alluvial diagram demonstrates the distribution overlap of the EC samples between risk score and molecular subtype (Figure S8D). CLUSTER 1 achieved a close ratio of two risk groups in the samples, while the distribution of CLUSTER 2 subtype samples was mainly confined to high risk scores. 

### 2.7. Expression Profile of Prognostic DEGs Clustered by Subtype and Clinical Factors 

To investigate the relative gene expressions of the four prognostic DEGs, the forward and reverse primer sequence of genes SRPX, IL6, HOXB9, and NR3C1 was shown as follows (Table 3). The mRNAs for the four genes were measured by real-time quantitative polymerase chain reaction (RT-qPCR). Endometrial tissues were treated with TRIzol reagent (Invitrogen, Waltham, MA, USA) for total-RNA extraction. Therefore, we explored the gene expressions of these four hypoxia-related genes in the subdivisions of the clinical categories as well as the total risk scores calculated compared between the subdivisions (Figure S9A). Expression of the prognostic genes were also compared between paratumor tissues and tumor tissues (Figure S9B). Summaries of differential expression of each prognostic DEG classified by age, histological type, grade, and stage was illustrated in the heatmap (Figure S10). 

**Figure 7:**
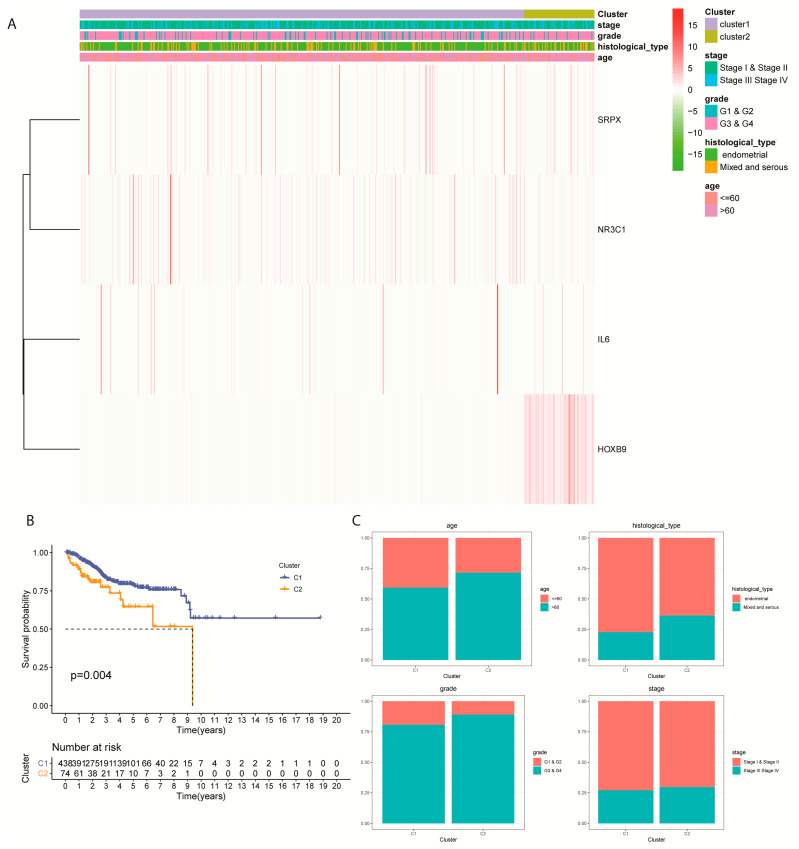
Main Clinical factors clustered into EC molecular subtypes. (**A**) Gene expression of four prognostic DEGs clustered by subtype and clinical factors. (**B**) Survival analysis of patients grouped by subtypes. (**C**) Barplots of survival probability of CLUSTER 1 and CLUSTER 2 in four main clinical factors (age, histological type, grade, and stage).

Furthermore, the EC samples obtained from the training cohort were classified into four typical clinical categories under each subtype (Figure 7A). The K-M OS curves of the two subtypes showed a significant prognostic difference in the TCGA-EC cohort that CLUSTER 1 has a higher survival probability compared to CLUSTER 2 (*p* = 0.004; Figure 7B). However, we observed no stage differences between CLUSTER 1 and CLUSTER 2, while these two molecular subtypes can be distinguished among survival probability patterns of age, histological type, and grade (Figure 7C).

### 2.8. Identification of Potential Targets for Immunotherapy and Chemotherapy in EC Molecular Subtypes

**Figure 8:**
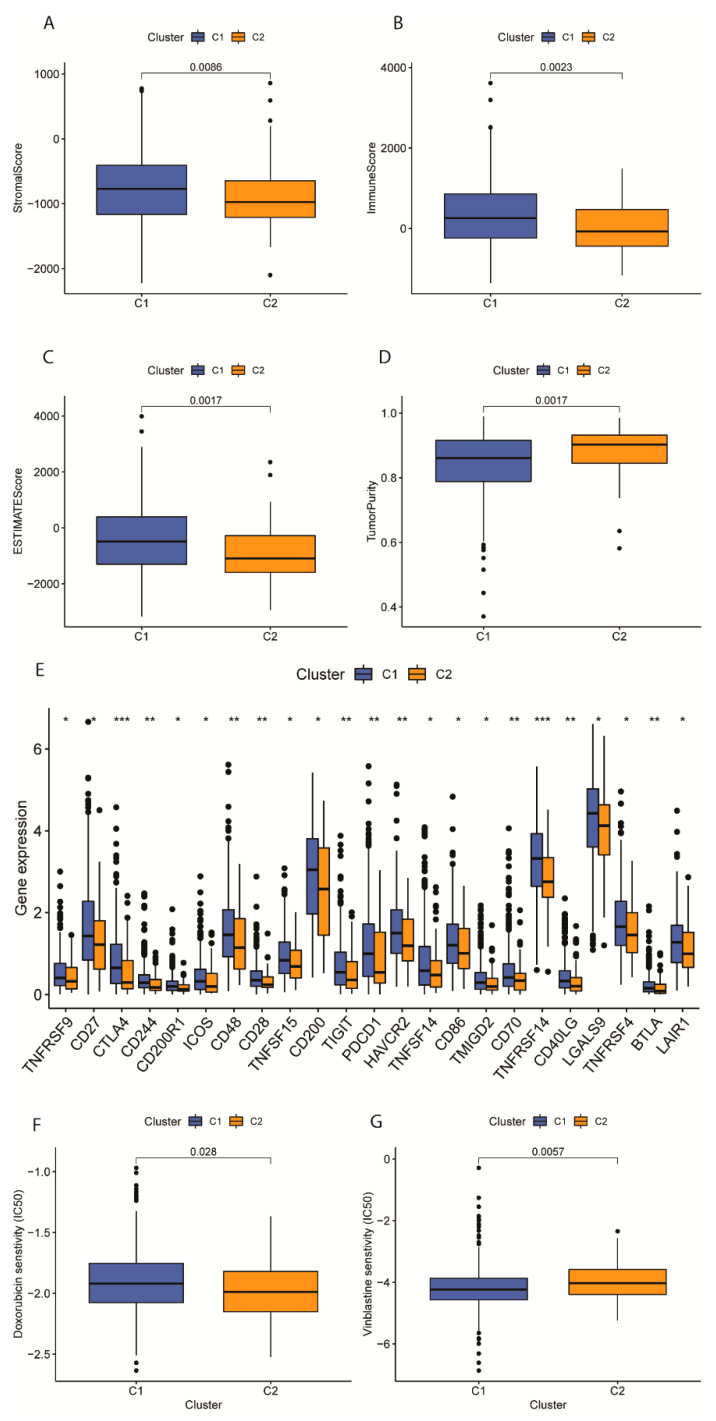
Tumor immune microenvironment analysis and drug sensitivity test for EC molecular subtypes. (**A**–**D**) Patterns of stromal cell scores, immune cell scores, ESTIMATE scores, and tumor purity between subtypes C1 and C2 of EC. (**E**) Expression profiles of 23 immune checkpoints between EC molecular subtypes. * means *p* < 0.05, ** means *p* < 0.01, *** means *p* < 0.001. (**F**) Box plot displaying the estimated IC50 values for Doxorubicin from the two molecular subtypes. (**G**) Box plot displaying the estimated IC50 values for Vinblastine from the two molecular subtypes.

We compared the amount of non-epithelial cells in each TCGA sample tissue between two molecular subtypes via the ESTIMATE algorithm. Patterns of Immune score, stromal score, and estimate score were increased in CLUSTER 1 with a decreasing Tumor purity (Figure 8A–D, *p* < 0.05). High immune and stromal scores indicated an enriched level of immune-reactive as well as mesenchymal expression [24]. Furthermore, low tumor purity indicates better prognostic outcomes. Several methods were used to compare the substantial differences in immune cell expressions in the two clusters (Figure S11).

To explore potential immunotherapy responses of patients under hypoxia, immune checkpoint expressions in different molecular subtypes were assessed. Classic differentially expressed immune checkpoints like CD27, CD70, CTLA4, and PDCD1 were included (Figure 8E). 

In terms of the chemotherapy, two drugs traditionally used in adjuvant chemotherapy for EC were evaluated for their subtype-specific sensitivity, respectively. A higher IC50 score of Doxorubicin was related to C1 (Figure 8F, *p* = 0.028). A lower IC 50 score of Vinblastine was related to Cluster 1 (Figure 8G, *p* = 0.0057).

### 2.9. A Nomogram Predicting Overall Survival for EC Patients by Subtype-Specific Signature and Clinical Factors

**Figure 9:**
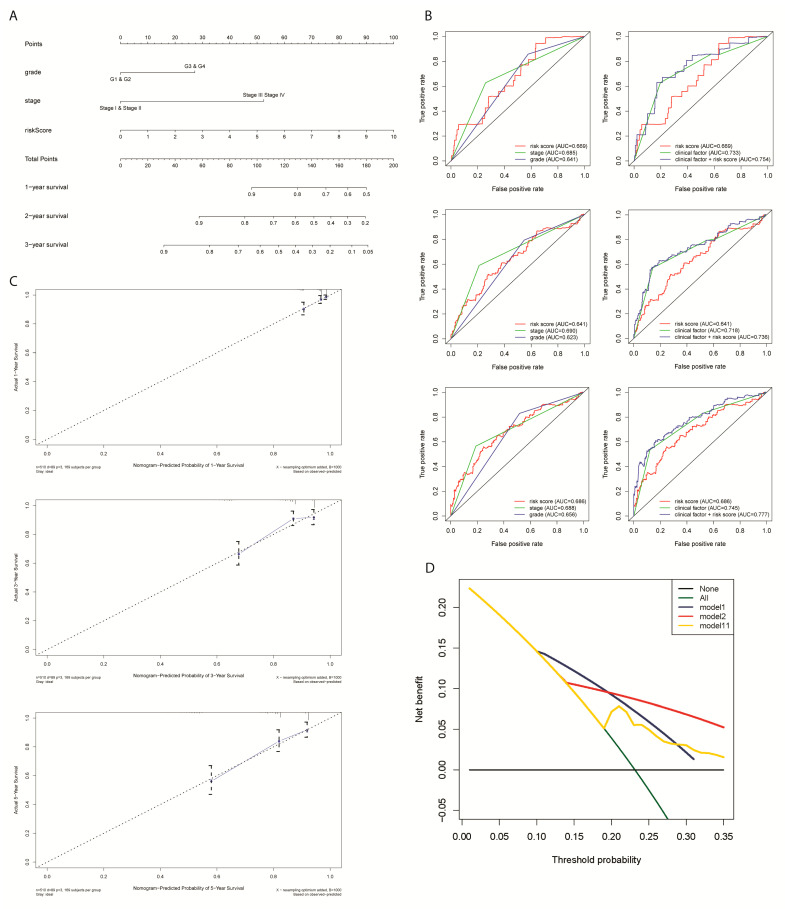
Predictive significance of signature verified in the nomogram model incorporating hypoxia-related gene signature and clinical characteristics. (**A**) Construction of a nomogram combining the four-gene signature and clinical features for the prediction of OS. (**B**) 1-,3-,5-year multi-ROC analysis for the final decision of the prognostic models. (**C**) Calibration plots displayed the actual and nomogram-predicted probability of one- (up), three- (middle), and five-year OS (down). (**D**) Decision curve analysis (DCA) curves of the nomogram for OS in HCC.

A nomogram was developed to further accurately predict the clinical outcomes by integrating the four-gene signature with two selected clinical characteristic variables (grade and stage) (Figure 9A). The prediction accuracy of prognostic models using risk score only and two independent clinical factors only or models using two clinical factors together and risk score with two clinical factors were compared by the multi-ROC analysis (Figure 9B). Additionally, calibration of the nomogram further validated a high consistency between the predicted survival probabilities of one-, three- and five-year OS and the observed data (Figure 9C). For this reason, the nomogram developed from our prognostic model should improve the prognostic result’s predictive power for EC patients compared to the previous signature model.

Importantly, the DCA plot showed no significantly higher net benefit between the risk score model, the clinical factor model, and the combined prognostic model (Figure 9D). Therefore, three models can be utilized under consideration of different applications.

## 3. Discussion

While histo-pathological tumor characteristics have been widely utilized for making clinical decisions in the past decade, the molecular subtype-based diagnostic and prognostic approaches are developing rapidly and have achieved advances in decision-making on targeted adjuvant therapies [32]. Some studies have pointed out that hypoxia commonly occurred during tumorigenesis and caused therapy resistance, which may influence the differentially expressed gene regulation in metabolic and immune systems [15,33]. In this study, we built a hypoxia gene signature to investigate the hypoxic TME’s association with tumor recurrence and to provide suggestions to both risk-based prognosis of EC patients and subtype-based diagnoses of EC progression.

Four DEGs (HOXB9, IL6, NR3C1, and SRPX) were selected as the predictor variables in the prognostic gene signature for an estimation of risk scores for the EC patients from the TCGA. According to our hypoxia-related gene signature, somatic gene alterations, especially PTEN mutation, were highly active regardless of a high or low risk score. During the development of EC in low grade, PTEN gene mutation is one of the most frequent mutations that tend to co-occur with PIK3CA and PIK3R1 gene mutations. Patients with PTEN mutation were susceptible to developing cancers like breast cancer, kidney cancer, and skin cancer [34]. Besides, loss of PTEN tended to cooperatively happen with CTNNB1 missense mutation and PIK3CA activation to boost myometrial invasion and thus form EC [35]. Another prominent mutation in our result was found at ARID1a, usually known as the tumor suppressor gene. It is reported that loss of ARID1a up-regulated PTEN in terms of the tumor cell proliferation in endometrial glands [36,37,38]. TP53 mutation is often associated with ECs in higher grades. However, the mutual occurrence of TP53 mutation and PTEN mutation is unique in USCs, which is closely related to our high-risk group [39,40,41]. Therefore, EC tumor cells in the hypoxic microenvironment gained specific somatic mutations including TP53 in high-risk patients, which promoted cell proliferation and lymph node metastasis. Nevertheless, the molecular mechanisms behind it were unclear and require further studies aided by animal models.

To look into the abnormalities at the transcriptional level, we explored the mRNA modification over the low and high-risk groups in EC samples. High level modifications in protein translation during the development of EC tumors were due to abnormal higher expression of m6A “readers” and “writers” [42,43]. Expressions of three m6A “readers”, YTHDF1, YTHDF3, and FMR1, as well as two m6A “writers,” KIAA1429 and WTAP, were enhanced in EC with predicted higher risks.

Gene ontology analysis disclosed a relationship between hypoxia-related DEGs and immune response, especially inflammatory, via regulations over macrophages or other cytokine receptors. One of the most active GO pathways in EC tissues was the response to the steroid hormone, which explained the effects of sex hormones interacting with insulin-like growth factors on EC tumorigenesis [44].

From the PCR results of the risk-predictor genes, we speculated high expression levels of NR3C1 in tumor tissues and expressions of IL-6 and SRPX converged in paratumor tissues. Previously known, amplification of HOXB9 in EC was correlated with poorer overall survival and EC progression [45]. IL-6, identified as a pro-inflammatory cytokine during inflammation and tumorigenesis, is related to microbial communities involved in immune responses [46]. NR3C1 was involved in the activation and enrichment of infiltrated immune cells including naive B cells, M1 macrophages, neutrophils, CD4 memory resting T cells, follicular helper T cells, gamma delta T cells, and regulatory T cells (Tregs) [47,48]. All four DEGs’ expressions were enhanced in high-risk EC patients.

Consistent with the previous studies, we also found that three out of four DEGs in hypoxia signature, SRPX, IL6, and HOXB 9 were positively associated with naive B cells, CD4 memory resting T cells, gamma delta T cells, M1 Macrophages, and resting Mast cells. Except for functions in innate immunity and autoimmunity, the immune cells mentioned were reported to have a role in modulating viral and bacterial [49,50]. CD4 memory resting T cells engaged in the secretion of C-X-C motif chemokine ligand (CXCL)10 during viral infection [49]. This interaction is consistent with a significantly higher expression of (CXCL)10 in high-risk EC patients. Aside from this, SRPX and IL6 were negatively correlated with regulatory T cells (Tregs). Tregs were recognized as a subset of T cells that suppresses immunity in sterilization and anti-tumor [51], thus SRPX and IL6 can be a novel target for recovering immune response in EC tumor cells. Moreover, Ryan, R et al. reasoned that SRPX also plays a part in the cell metabolism relating to the glucocorticoid GO pathway [52]. Consequently, our prognostic signature model could precisely predict the potential risk of EC patients by detecting aberrancies in the genome and proteomes.

Surprisingly, gathering results of ESTIMATE scores, high profiles of stromal cells and unique neuroendocrine-like immune cells bridged our hypoxia signature to an immune- desert molecular subtype of ovarian cancer determined by quantitative immune phenotypes [53]. “Cold” tumor subtype was defined as scarce CD8+T cells and identified in pancreatic, gastric, and ovarian cancer [54,55,56]. In details, a smaller number of T cells driven by CD8 were associated with higher risk in EC patients. TME of desert subtype explored in pancreatic cancer was proved to have enriched B cell expression [54], which was also supported by infiltrated neuroendocrine-like immune cell including human-activated dendritic cell (aDC) and B cell enrichments in the EC high-risk group. In Zhang B et al. study, the desert subtype was found consistent with the m6a modification patterns, which confirmed that desert sub-TMEs has a close relationship with immune ignorance and loss of T cells [55]. KIAA1429, among all m6a regulators, were positively associated with sub TMEs of desert or non-infiltrated subtype in gastric cancer, which is consistent with the high-risk group predicted with hypoxia signature. Noticeably, either desert-dominant or co-occurred subtype was correlated with a poorer survival outcomes [55]. However, further identification including the definitive measurement and spatial distribution of CD8+ T-cell versus stromal cells were required.

Standard treatments on EC are confined to excision surgeries on the lesion [54,57]. Along with the deepened insight into EC’s staging and histological classifications, patients diagnosed with higher grades will be treated with systemic therapies mixed with radiotherapy, and low-grade will be treated with chemotherapy and targeted therapies for trials. Several chemo-drugs were previously studied regarding EC treatments [58]. Gebbia V et al. tested a combination including Cisplatin and Vinorelbine and insisted that the regimen was preferable to the classical Anthracycline [59]. Another drug, Docetaxel was reported to have considerable efficacy and a bearable range of toxicity in two phase II trials [51,60]. The significant chemotherapeutic sensitivity of Vinorelbine and Docetaxel in the high-risk group proved their fit for patients with high risk even encountering the hypoxia-raised therapy resistance. 

Emerging targeted medications include ICI anti-PD-1/PD-L1 and chemotherapy [35,61]. PD-L1 and PD-L2 displayed positive relationships with hypoxia DEGs prognostic risks, which indicated anti-PD-1/PD-L1 and anti-PD-1/PD-L1 treatment should be a good choice explicitly targeting the predicted high-risk group. Present studies further supplemented that increasing number of mutations gave rise to a growing class of neo-antigens, which could be targets for T-cell attack [62,63]. Tumor mutation burden was therefore considered an index of patients’ response to immunotherapy [64,65]. We figured out a higher TMB for low risk group indicating higher immunotherapy benefit by targeting various checkpoints in terms of advanced prevention, although PD-1/PD-L1 immunotherapy was effective in the high risk group.

Two molecular subtypes were characterized upon the hypoxia gene expressions. An overlap between distributions of molecular subtype and risk groups was discovered in TCGA dataset. CLUSTER 2 was majorly high-risk, while CLUSTER 1 had a subtly higher ratio distributed in low-risk than high-risk. Significantly higher immune score, stromal score and estimate score calculated on CLUSTER 1 subtype explained the abundance of immune cells and stromal cells in tumor tissues. CLUSTER 1 was also estimated with a lower tumor purity, which suggested a less complex tumor microenvironment and better clinical result. Our chemotherapeutic sensitivity results also revealed relatively significant effects of Vinorelbine over CLUSTER 1, compatible with results of high-risk patients. As a result, subtype-based diagnosis recommended surgery and targeted chemotherapy on patients in CLUSTER 1, while more personalized medications on patients in CLUSTER 2.

The hypoxia gene signature was eventually coupled with clinical factors, which were selected by the univariate and multivariate hazard analysis, to build a prognostic model. We constructed a nomogram for the model, which was validated for the improved predictive power and thus was recommended for EC prognosis.

Due to the research subjects’ particularity, and ethical reasons, our study still had some limitations. Firstly, although a large sample size and quality control were obtained, data resources were limited to TCGA, and more evidence of hypoxia signature from other databases can be sought for confirmation. Secondly, as a cross-sectional retrospective study without longitudinal follow-up data, we cannot confirm whether TME changes occurred after the recorded histology and stages. However, our model showed a superiority over other prognostic models solely comprising gene signature. Additionally, we have provided some auxiliary analysis regarding m6A regulators and cancer cell stemness, which can be supportive evidence for cancers progressions associated with metabolic reprogramming. 

Recent studies have found several gene mutations, including PTEN, TP53, PIK3CA, and classified four major genomic classes of endometrial cancer. However, the scope of prominent mutations can be narrowed to identify the target with the most direct relationship with tumor cell growth and cancer progression. Besides, our study proposed a possible relationship with immune-desert subtype. Future investigation can be directed to the differential responses to chemotherapy and immune checkpoint inhibition for three immune subtypes with the consideration of hypoxic TME.

## 4. Methods and Materials

### 4.1. Data Collection and Preprocessing

There were 200 hypoxia genes chosen from The Molecular Signatures Database v7.2 (https://www.gsea-msigdb.org/gsea/msigdb, “HALLMARK_HYPOXIA”, accessed on 3 May 2022), which were found to be up-regulated in hypoxic condition via Gene Set Enrichment Analysis v4.1.0 software [66].

We retrieved the data regarding somatic mutations and clinical factors (age, histological type, grade, stage, and survival information) for EC from the TCGA public database (http://cancergenome.nih.gov/, accessed on 6 May 2022). Therefore, 512 EC samples from TCGA were randomly divided into the training and testing cohort with an approximately 1:1 ratio. The hypoxia-related differentially expressed genes (DEGs) were filtered by the linear models for microarray data with FDR < 0.001 using the R limma package.

### 4.2. Functional Annotation Analysis

Kyoto Encyclopedia of Genes and Genomes (KEGG) pathway and Gene Ontology (GO) enrichment analyses were performed on hypoxia-associated DEGs between high and low-risk cohorts by the R clusterProfiler package [21]. Gene enrichment analysis includes three GO terms, biological process (BP), molecular function (MF), and cellular component. GO terms and KEGG pathways were considered statistically significant with *p* < 0.05.

### 4.3. Establishment of a Hypoxia Gene Signature

To explore the prognostic significance of the 29 DEGs relating to hypoxia in EC, a univariate Cox regression analysis was performed. Five prognosis-related DEGs with *p* < 0.05 were screened in the training dataset. After the LASSO Cox regression analysis by the glmnet R package [67], four out of five hypoxia-related DEGs were identified (Figure S2). A multivariate Cox regression was performed to formulate a hypoxia-related gene combination (Table S2). 

### 4.4. Formulation and Validation of a Nomogram for the Prognostic Model

We further integrated the risk score evaluated by the hypoxia gene signature and the clinical factors into a nomogram facilitating the OS prediction by the R rms package [68]. Based on the nomogram model, the total survival probability of each patient can be calculated by summarizing the corresponding points of all variables. Calibration plots of the nomogram were used to illustrate the fitness of the predicted 3-, 5-, and 10-year survival compared to the observed value. The decision curve analysis (DCA) was used to check the predictive power.

### 4.5. Quantitative Real-Time Polymerase Chain Reaction PCR after the RNA Isolation

The research was approved by the First Affiliated Hospital of Nanjing Medical University Ethics Committee. The participants entered the research cohort strictly provided us with written informed consent. RNA was extracted from 15 EC and 15 normal sample tissues with TRIzol reagent (Thermo Fisher Scientific, Waltham, MA, USA), and complementary DNA (cDNA) was synthesized using the total RNA via the high-capacity reverse transcription kits (TaKaRa, Shiga, Japan) (Table S3). Assays were used to perform the RT-qPCR based on SYBR Green PCR Kit (Thermo Fisher Scientific, Waltham, MA, USA). The 2^−ΔΔCT^ method was applied on Light Cycler 480 (Roche, Basel, Switzerland). The forward and reverse primer sequences used in qRT-PCR are listed in Table 3. 

### 4.6. Genomic Alteration Analysis

We analyzed the gene variations from the Genomic Identification of Significant Targets in Cancer v2.0 by the software, genePattern. Specifically, frequencies of somatic mutations were calculated using the MutSigCV algorithm [69]. Moreover, we plotted the TMB score for EC patients from the TCGA dataset to predict the immunotherapeutic impacts for patients with varying risk scores. To determine the disparities in TMB levels, the Wilcoxon rank sum test was employed.

### 4.7. Identification of Immune Cell Types and Assessment of Significant Immune Checkpoint Inhibitors 

The infiltration levels of twenty-two kinds of immune cells were calculated utilizing the Cell type identification by estimating relative subsets of RNA transcripts (CIBERSORT) algorithm. We calculated immune infiltration variations between two molecular subtypes through the Wilcoxon rank-sum test [70]. Besides, the correlation of the immune cell types with hypoxia gene signature was evaluated with absolute value and *p* < 0.05. 

### 4.8. Estimation of Immune and Stromal Cells in EC

Using expression profiles from TCGA samples, we evaluated infiltrating stromal and immune cell levels in EC diseased cells by the Estimation of stromal and immune cells in malignant tumors using the expression data (ESTIMATE) algorithm. By pooling stromal and immune scores, the ESTIMATE score was subsequently evaluated. The tumor purity of samples from each TCGA patient was then determined for the corresponding ESTIMATE scores [24]. 

### 4.9. Expression Analysis of m6A RNA Methylation Regulators

According to recent papers, we chose twenty m6A RNA methylation regulators (METTL3, HNRNPC, YTHDC1, ZC3H13, YTHDF2, FTO, YTHDF1, YTHDF3, YTHDC2, METTL14, RBM15, WTAP, KIAA1429, FMR1, METTL16, HNRNPA2B1, and ALKBH5) for further research. Using the CIBERSORT algorithm, the risk differences in expression profiles of m6A regulators were compared for the EC tissues in TCGA samples with *p* < 0.001. 

### 4.10. Chemotherapy Sensitivity Test

To explore substantially different responses of chemotherapeutic drugs in the molecular subtypes of EC and risk groups predicted by the hypoxia gene signature model, we calculated the IC50 of drugs typically applied in regiments of ECs using the R pRRophetic package. The sensitivity response of each patient in chemotherapy was predicted by database extracted from the Genomics of Drug Sensitivity in Cancer (GDSC; https://www.cancerrxgene.org/, accessed on 7 May 2022) [71]. 

### 4.11. Clustering Analysis

The consistent clustering identified molecular subtypes of EC samples from TCGA via the ConsensusClusterPlus package in R software [72]. In the clustering analysis, we used transcriptomic profiling data of four hypoxia-related signature genes, survival time, survival status, predicted risk score, and risk groups as dimensions of each sample. Then, we calculated the Euclidean squared distance metric and the K-means clustering algorithm from k = 2 to k = 9. Besides, we performed the principal components analysis (PCA) and t-distributed stochastic neighbor embedding (t-SNE) to perform multiples tests on clustering results established on the transcriptome expression profile of the above hypoxia-related genes.

### 4.12. Statistical Analysis

We predominantly performed data analysis with the aid of the R language v4.0.2 software throughout the study (https://www.r-project.org/, accessed on 7 May 2022). Different hypoxia subtypes were compared by the Kruskal–Wallis test. The differential survival time was figured out using the log-rank test with *p* < 0.05 and we applied Kaplan Meier curves to illustrate the striking distinctions in survival time.

## 5. Conclusions

We portrayed two hypoxia-related molecular subtypes of EC based on the four screened DEGs signature, which integrated with clinical factors to serve as a predictive model for EC patients. The assessments on infiltrating immune cell types, immune checkpoint inhibitors, and chemotherapy responses can be referred to as some insights into the hypoxic impacts of the genome, methylome, and transcriptome on EC progression in the future.

## Figures and Tables

**Table 1:** Univariate and multivariate Cox model result of the training set, testing set, and entire set.

Variable	Univariate Cox Model				Multivariate Cox Model			
	HR	HR.95L	HR.95H	*p* Value	HR	HR.95L	HR.95H	*p* Value
Training set								
age	2.3010	1.1898	4.4499	0.0133	2.2176	1.1146	4.4121	0.0233
stage	3.6435	2.0219	6.5656	0.0000	3.3310	1.7492	6.3431	0.0003
histological_type	2.0604	1.1423	3.7165	0.0163	0.9318	0.4581	1.8952	0.8454
grade	2.1739	1.1239	4.2049	0.0211	1.3027	0.6030	2.8143	0.5010
riskScore	1.3662	1.1800	1.5817	0.0000	1.2244	1.0440	1.4360	0.0128
Testing set								
age	1.3332	0.6821	2.6061	0.4003	0.9553	0.4598	1.9848	0.9025
stage	4.7100	2.5689	8.6357	0.0000	2.8626	1.5063	5.4402	0.0013
histological_type	4.6591	2.5439	8.5331	0.0000	2.0849	1.0231	4.2483	0.0431
grade	7.5966	2.7083	21.3077	0.0001	3.9697	1.3121	12.0107	0.0147
riskScore	1.3974	1.1728	1.6649	0.0002	1.1233	0.9240	1.3657	0.2433
Entire set								
age	1.7782	1.1121	2.8432	0.0162	1.5240	0.9300	2.4974	0.0946
stage	4.1162	2.7000	6.2754	0.0000	3.0942	1.9669	4.8676	0.0000
histological_type	3.0435	2.0032	4.6242	0.0000	1.3605	0.8292	2.2323	0.2231
grade	3.3973	1.9765	5.8397	0.0000	1.9294	1.0493	3.5478	0.0345
riskScore	1.3956	1.2489	1.5596	0.0000	1.1991	1.0610	1.3552	0.0036

**Table 2:** The expression difference of immune checkpoints between low and high-risk groups.

Gene	*p*-Value
IDO1	0.0326
CD27	0.2540
CD58	0.5882
CTLA4	0.3644
ICOS	0.0129
PD-L2	0.0000
B7-H3	0.0153
B7-H4	0.9876
TIGIT	0.1388
PD-1	0.3375
CD40	0.0000
LAG3	0.0061
TIM-3	0.1870
CD86	0.0214
PD-L1	0.0023
CD70	0.1660
CD270	0.0239

**Table 3:** PCR results of four hypoxia-related genes in the EC prognostic model.

Primer	Primer Sequence (5’ to 3’)	Base Pairs
SRPX F	ATCAAGGTGAAGTATGGGGATGT	23
SRPX R	GTTTGACTGGCAGATCAGTAGG	22
IL6 F	ACTCACCTCTTCAGAACGAATTG	23
IL6 R	CCATCTTTGGAAGGTTCAGGTTG	23
HOXB9 F	CCATTTCTGGGACGCTTAGCA	21
HOXB9 R	TGTAAGGGTGGTAGACGGACG	21
NR3C1 F	ACAGCATCCCTTTCTCAACAG	21
NR3C1 R	AGATCCTTGGCACCTATTCCAAT	23

## Data Availability

The datasets supporting the conclusions of this article are included within the article. Public datasets in this study were available for additional analysis. Datasets needed for reproducibility can be retrieved from “HALLMARK_HYPOXIA” repository, the TCGA website [http://cancergenome.nih.gov/&lt;/b&gt] (accessed on 3 May 2022). Data analysis was performed using the R language v4.0.2 software throughout the study (https://www.r-project.org/, accessed on 3 May 2022).

## Supplementary Materials

The following supporting information can be downloaded at: https://www.mdpi.com/article/10.3390/ijms24021675/s1.

## Abbreviations

aDCs	human-activated dendritic cells
CIBERSORT	Cell type identification by estimating relative subsets of RNA transcripts
DEGs	differentially expressed genes
EC	endometrial carcinoma
ESTIMATE	Estimation of stromal and immune cells in malignant tumors using expression data
GDSC	Genomics of Drug Sensitivity in Cancer
GSVA	Gene set variation analysis
GO	Gene Ontology
HR	hazard ratio
ICI	immune checkpoint inhibitors
IC50	the semi-inhibitory concentration
iDCs	immature dendritic cells
KEGG	Kyoto Encyclopedia of Genes and Genomes
LASSO	least absolute shrinkage and selection operation
Limma	linear models for microarray data
m6a	N^6^-methyladenosine
OS	overall survival
PCA	principal components analysis
ROC	receiver operating characteristic curves
TCGA	The Cancer Genome Atlas
TMB	tumor mutation burden
Tregs	regulatory T cells
t-SNE	t-distributed stochastic neighbor embedding
