# Supplementary material for: A Hypoxia Molecular Signature-Based Prognostic Model for Endometrial Cancer Patients
Source: Int J Mol Sci. 2023 Jan 14;24(2):1675. doi: 10.3390/ijms24021675 (PMC9866886; doi:10.3390/ijms24021675)
Supplement: Supplementary file 1 [file ijms-24-01675-s001.zip › ijms-2078841-supplementary.pdf]

Table S1. 29 hypoxia-related DEGs screened with $p < 0.001$ from TCGA-EC cohort ($n = 512$)

gene	conMean	treatMean	logFC	pValue	fdr
BRS3	0.2725	0.0342	-2.9931	0	0
PKP1	0.0924	1.4776	3.9998	0.0015	0.0023
EFNA3	0.8702	6.441	2.8879	0	0
CAV1	110.0484	8.6215	-3.674	0	0
SRPX	23.3521	2.1627	-3.4327	0	0
B4GALNT2	0.002	0.1021	5.7013	0	0
DUSP1	522.0268	50.8053	-3.3611	0	0
FOS	538.2365	69.1391	-2.9607	0	0
HAS1	1.1086	0.1068	-3.3753	0	0
GCNT2	0.9297	4.471	2.2658	0	0
STC2	1.2055	10.9693	3.1857	0	0
CP	6.71	32.2913	2.2668	0	0
SLC2A1	13.6979	55.9282	2.0296	0	0
ATF3	52.91	12.1715	-2.12	0	0
IL6	7.5606	1.829	-2.0474	0.0012	0.0019
HOXB9	0.6679	26.0854	5.2875	0	0
CAVIN1	283.3312	32.9774	-3.1029	0	0
PPFIA4	0.1859	1.1179	2.5885	0	0
PYGM	5.1116	0.6374	-3.0036	0	0
DCN	163.0059	7.8145	-4.3826	0	0
NR3C1	9.1646	1.5147	-2.5971	0	0
ZFP36	368.0634	75.7327	-2.281	0	0
BCAN	1.726	0.4189	-2.0428	0	0
ADM	4.4513	21.9337	2.3008	0	0
AKAP12	26.1425	5.4633	-2.2586	0	0
EDN2	1.363	6.4343	2.239	0	0
SULT2B1	0.3044	7.0829	4.5404	0	0
PPP1R3C	11.969	1.0496	-3.5114	0	0
CITED2	111.1252	16.7127	-2.7332	0	0

Table S2. Multivariate Cox regression analysis result

id	coef	HR	HR.95L	HR.95H	pvalue
SRPX	0.0625	1.0645	1.0120	1.1197	0.0154
IL6	0.0162	1.0163	1.0033	1.0295	0.0140
HOXB9	0.0061	1.0061	1.0011	1.0111	0.0157
NR3C1	0.1555	1.1682	0.9916	1.3764	0.0631

Table S3. Patients' clinical characterization diagnosed with endometrial cancer in the RT-qPCR test (compared with 15 normal samples as control set)

ID	Age	Grade	Stage	Histology
1	53	I	IA	Endometrial adenocarcinoma
2	46	II	IA	Endometrial adenocarcinoma
3	56	II	IA	Endometrial adenocarcinoma
4	45	I - II	III	Endometrial adenocarcinoma
5	64	II - III	IA	Endometrial adenocarcinoma
6	63	II	IA	Endometrial adenocarcinoma
7	62	I	IA	Endometrial adenocarcinoma
8	53	II	IIIB	Endometrial adenocarcinoma
9	69	II	IA	Endometrial adenocarcinoma
10	56	II	IA	Endometrial adenocarcinoma
11	62	II	IB	Endometrial adenocarcinoma
12	58	II	IA	Endometrial adenocarcinoma
13	61	II	IA	Endometrial adenocarcinoma
14	46	II	IA	Endometrial adenocarcinoma
15	48	II	IA	Endometrial adenocarcinoma

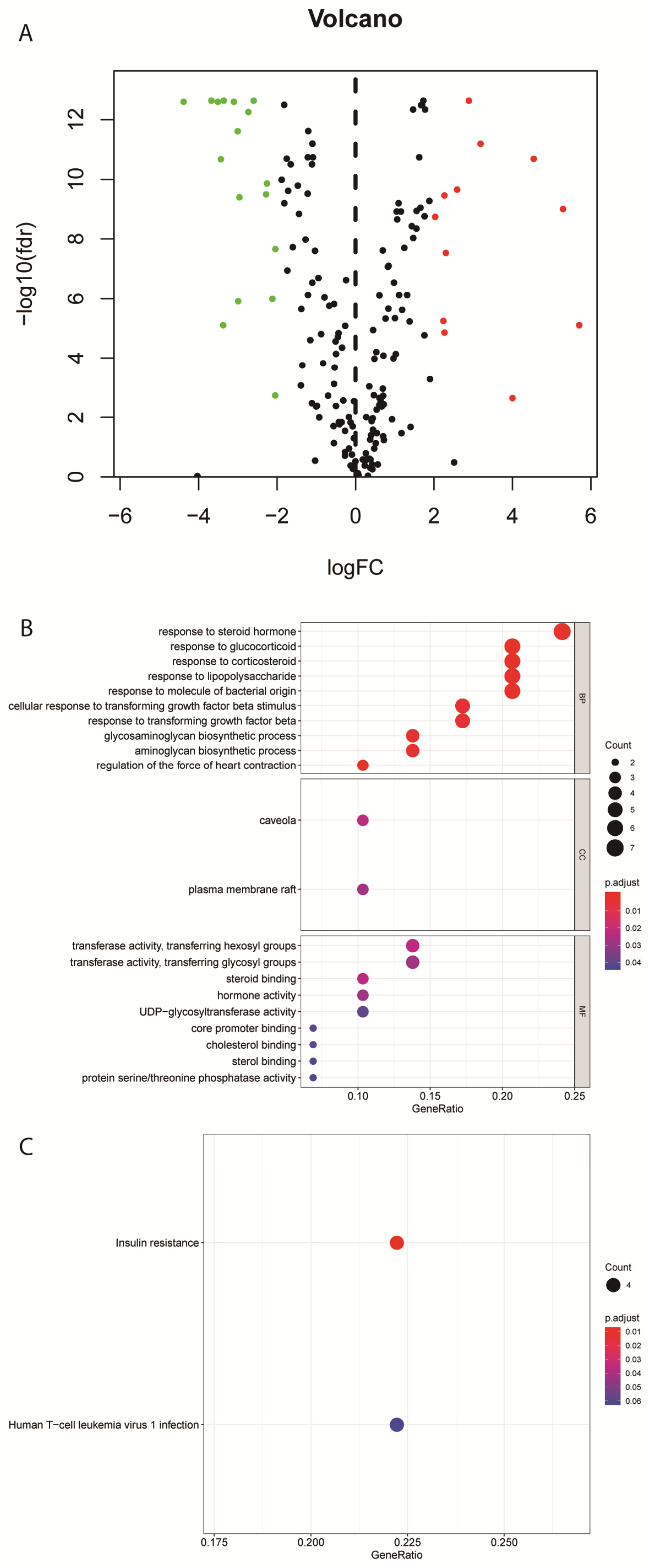

Figure S1. (A) Volcano plot of differentially expressed hypoxia-related genes in the TCGA dataset (B) Bubble plot of GO functional pathways including biological processes (BP), cellular component (CC) and molecular function (MF) categories. Red indicates high enrichment and blue indicates low enrichment (C) Bubble plot of two significantly enriched KEGG signaling pathways

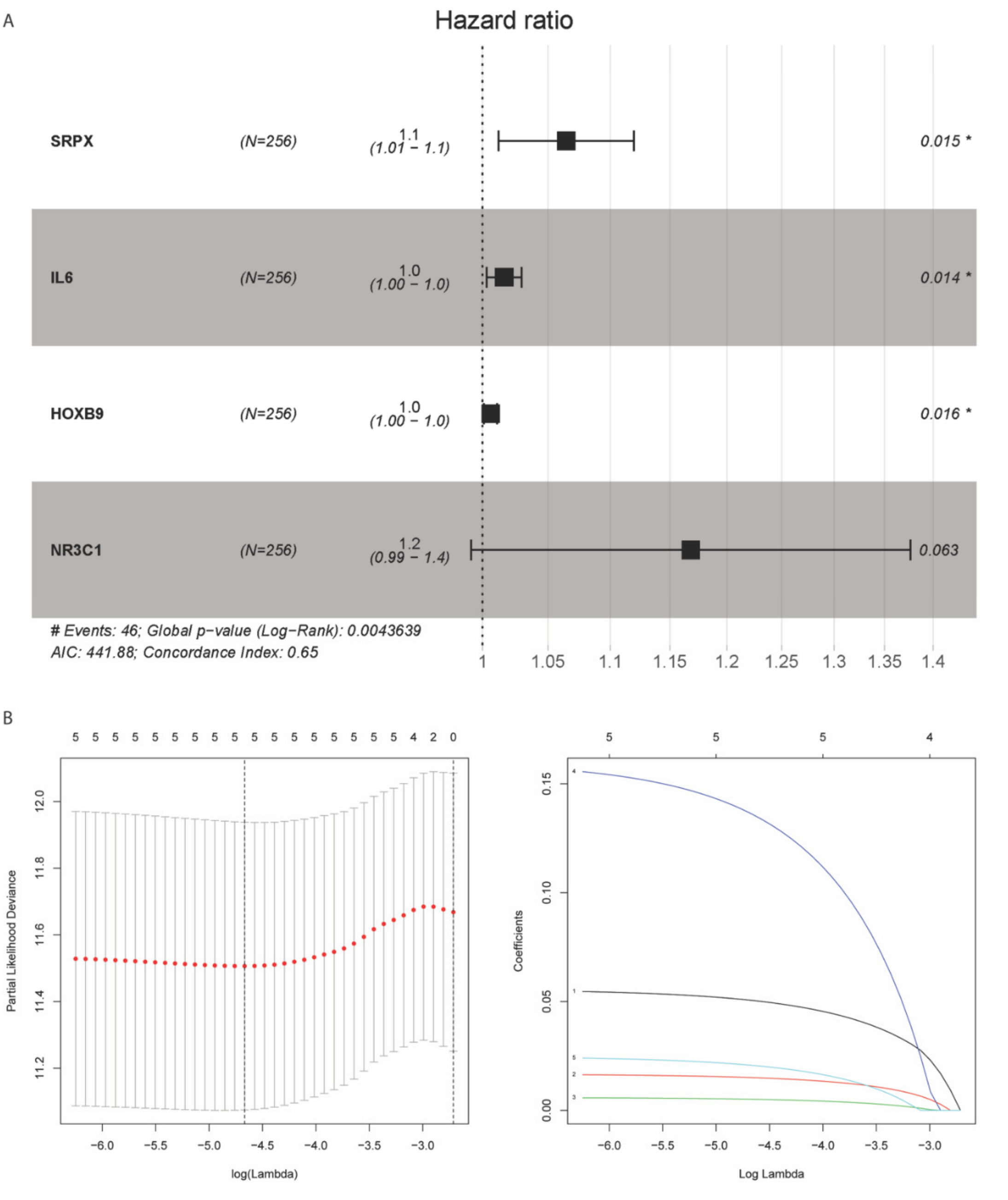

Figure S2. (A) Forest plot of hazard ratio calculated for four prognostic DEGs. (B) Plots of the LASSO coefficients (left), Adjustment of parameter selection in LASSO-Cox analysis with 20-time cross-validation (right);

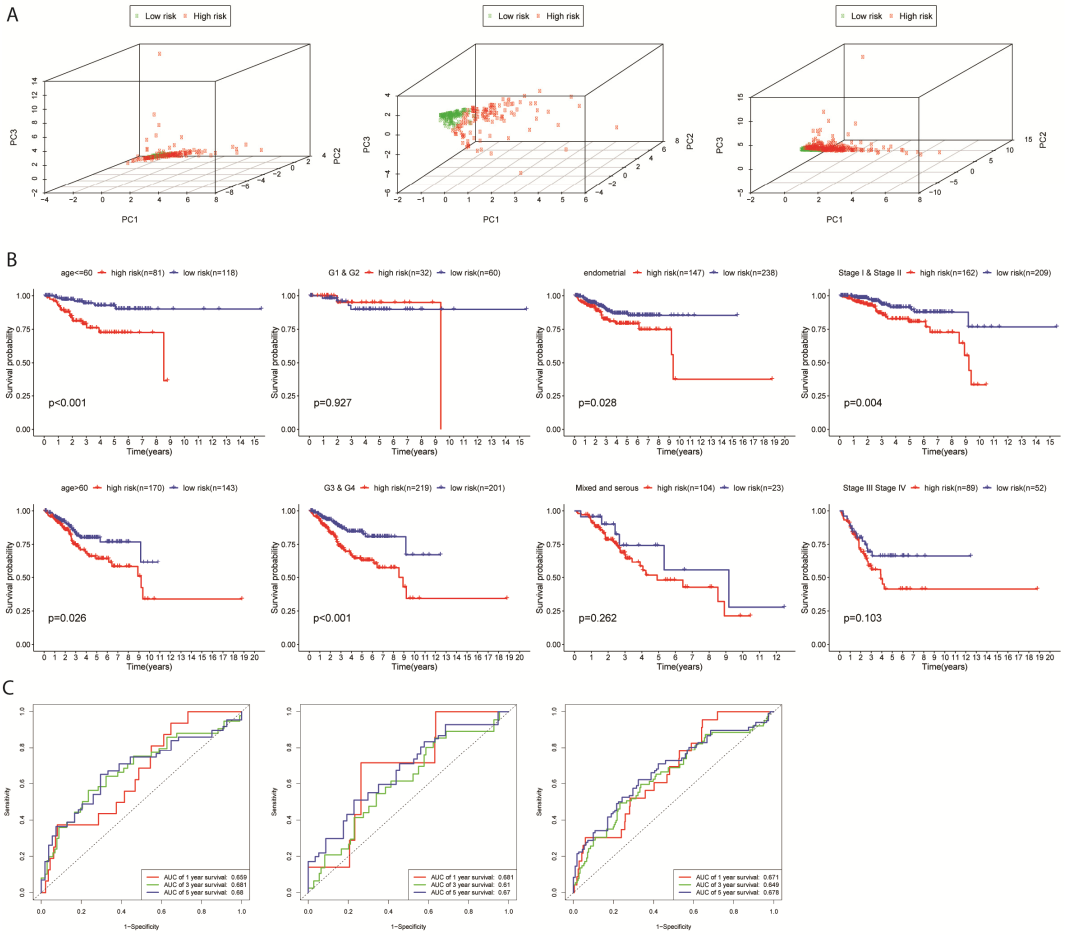

Figure S3. (A) Principal Component Analysis (PCA) plot of training (left), testing (center), and entire set (right) in TCGA; (B) Survival probability of four clinical factors (age, grade, stage, and histological types) in subdivisions compared between low (blue) and high-risk (red) groups (C) Time-dependent ROC curves for one-, three- and five-year OS time in the training set, test set, and entire set.

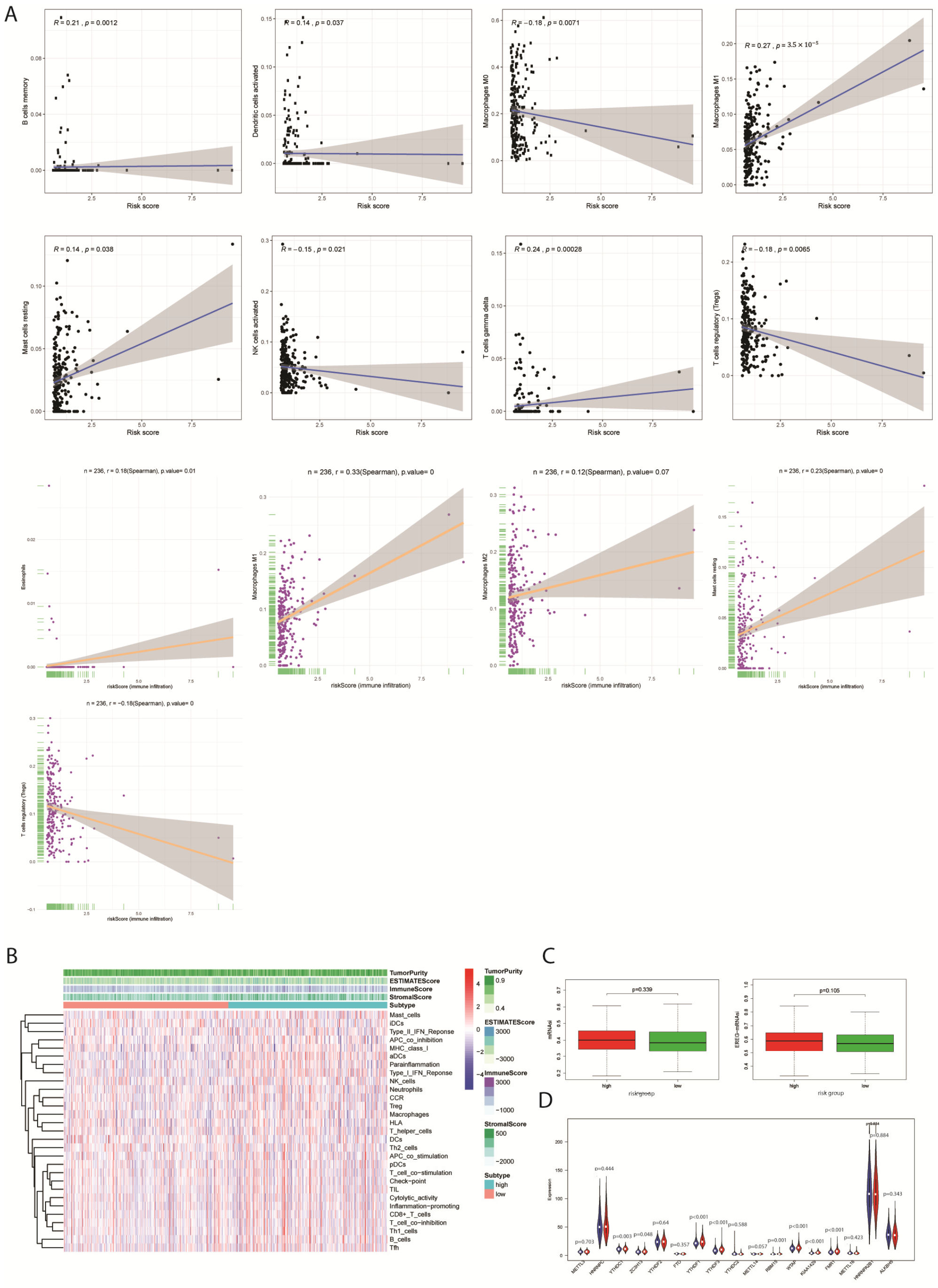

Figure S4. (A) Associations of immune cell infiltration level with the risk score in TCGA; Blue: Correlations between risk score and expression of eight immune cell types. Orange: Correlations between the risk model and infiltration abundances of six types of immune cells; (B) Clustered expression profile of non-epithelial cells in EC samples grouped into molecular subtypes, stromal score, immune score, ESTIMATE score, and tumor purity. Red for highly up-regulation; Blue for highly down-regulation. (C) mRNA expression-based stemness index (mRNAsi) and epigenetically regulated mRNAsi (EREG-mRNAsi) calculated for low and high-risk groups in EC patients (D) The expression difference of RNA methylation regulator (m6A) between low and high-risk groups in TCGA-EC datasets. Red represents the high-risk group; blue represents the low-risk group.

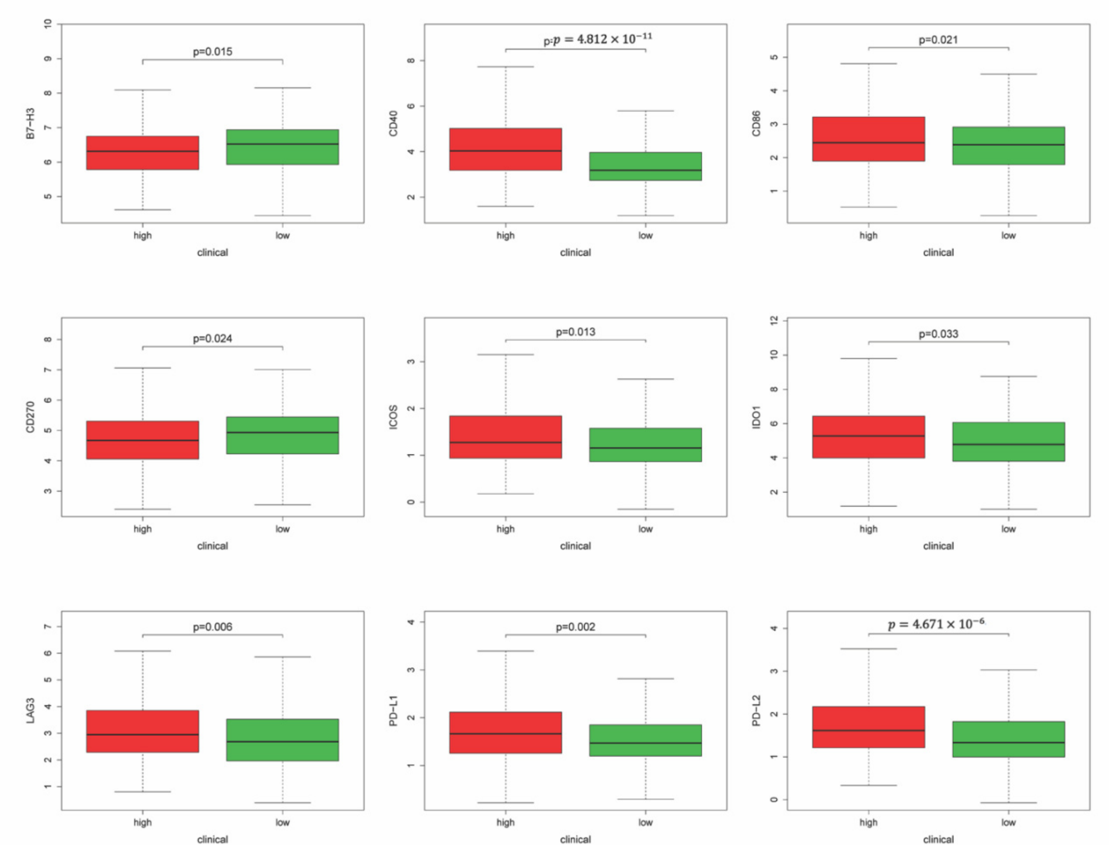

Figure S5. Boxplot of immune checkpoints' expression differentiated in the low and high-risk group

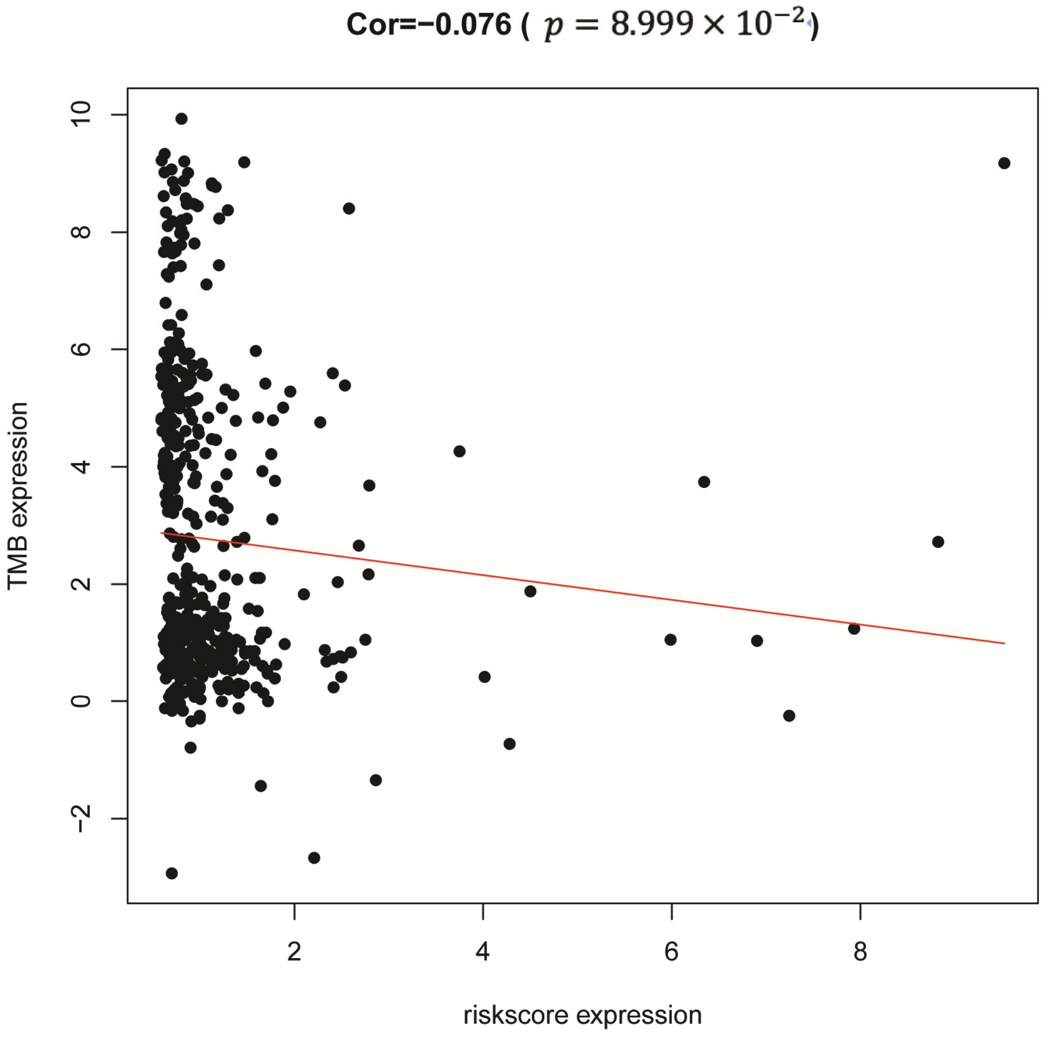

Figure S6. The relationship between the hypoxia-related risk signature and TMB

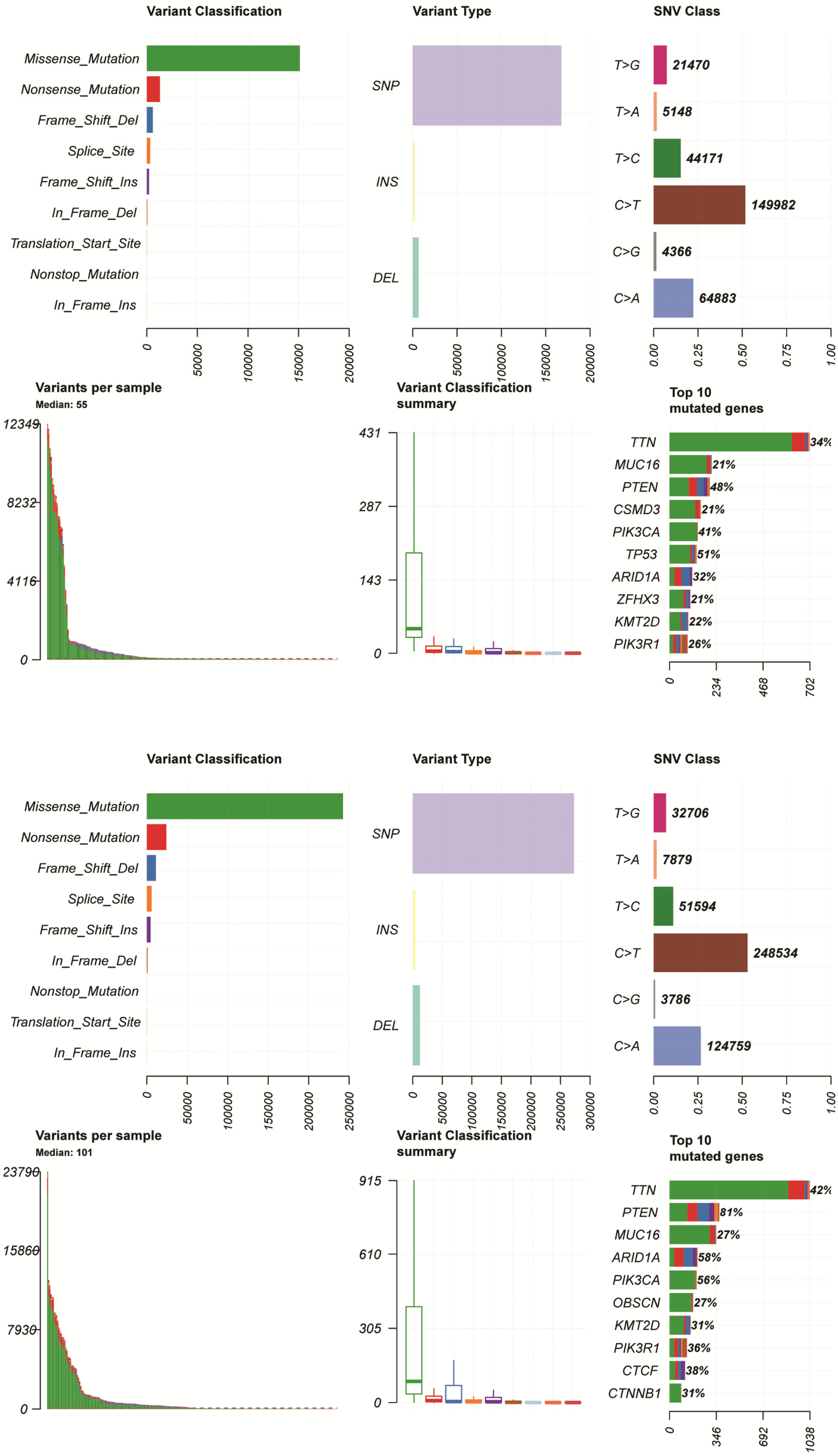

Figure S7. Summaries of gene alterations in TMB analysis

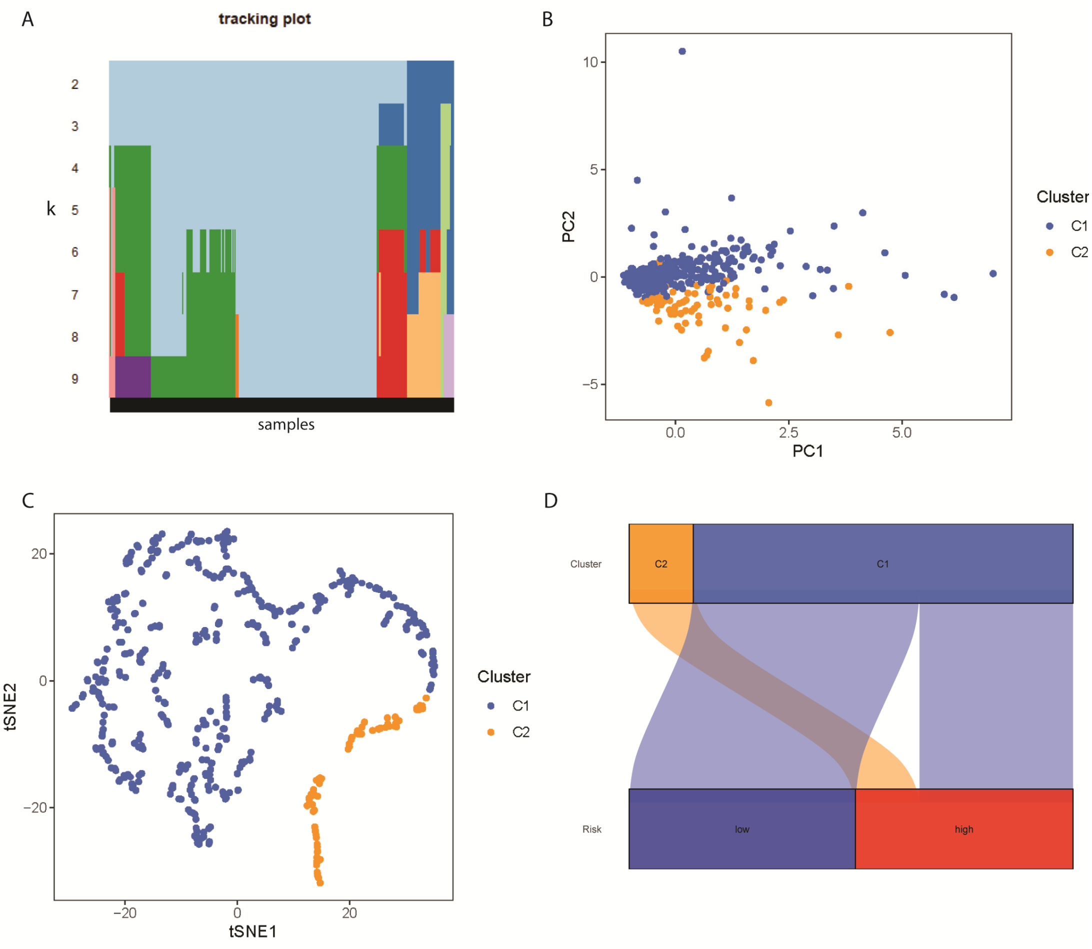

Figure S8. (A) Distribution of samples according to cluster index from k=2 to =9; (B) Principal Component Analysis (PCA) plot showing a difference in transcriptomes between the two subtypes (left); (C) Alluvial diagram of risk group distributions in molecular subtypes of EC; (D) t-distributed stochastic neighbor embedding (t-SNE) clustering of gene expression in EC (right)

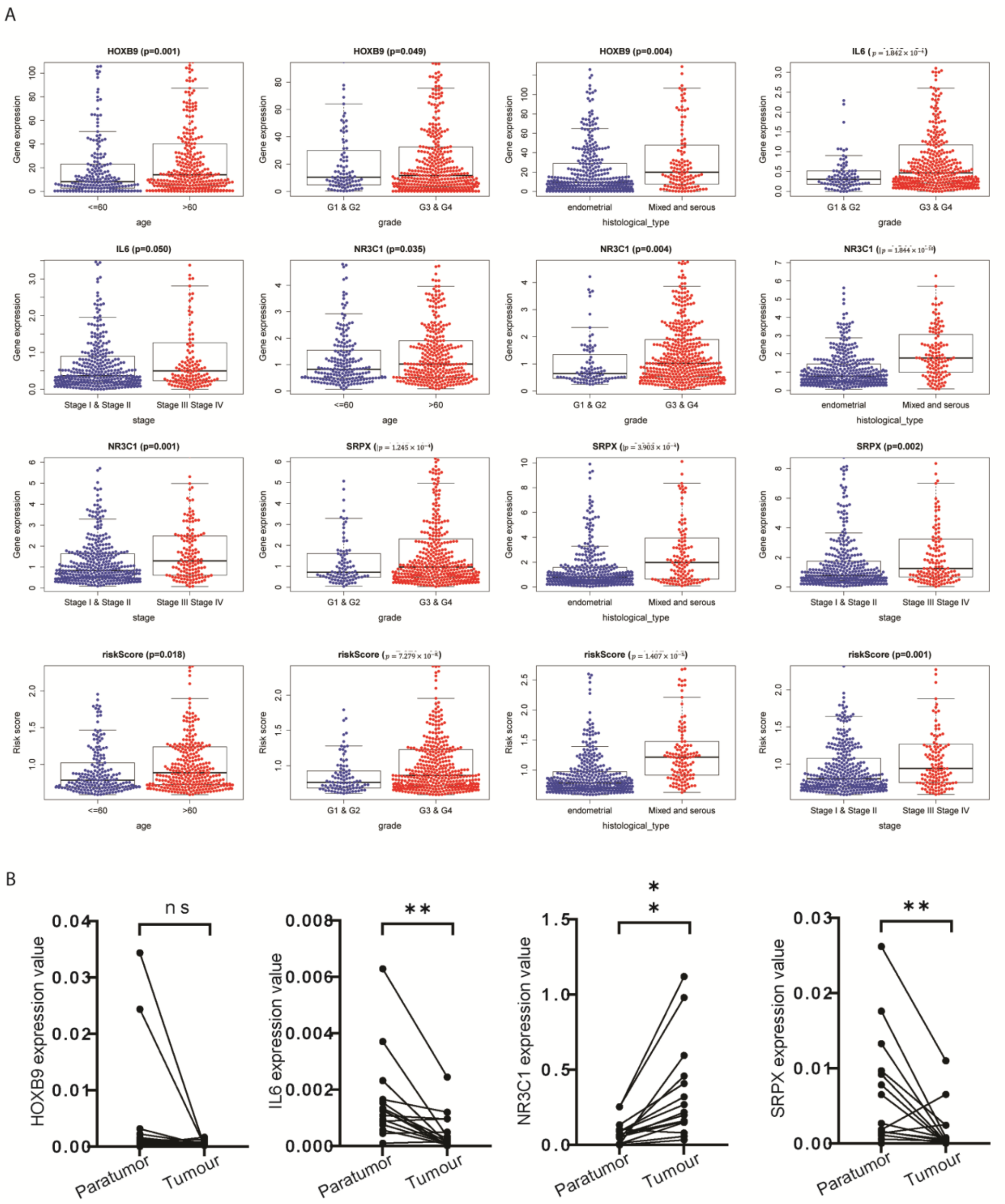

Figure S9. (A) Gene expressions of four genes in the EC hypoxia signature compared between subdivisions of the clinical factors (age, grade, stage, and histological types). (B) Comparisons between expression levels of Prognostic DEGs in paratumor and tumor tissues.

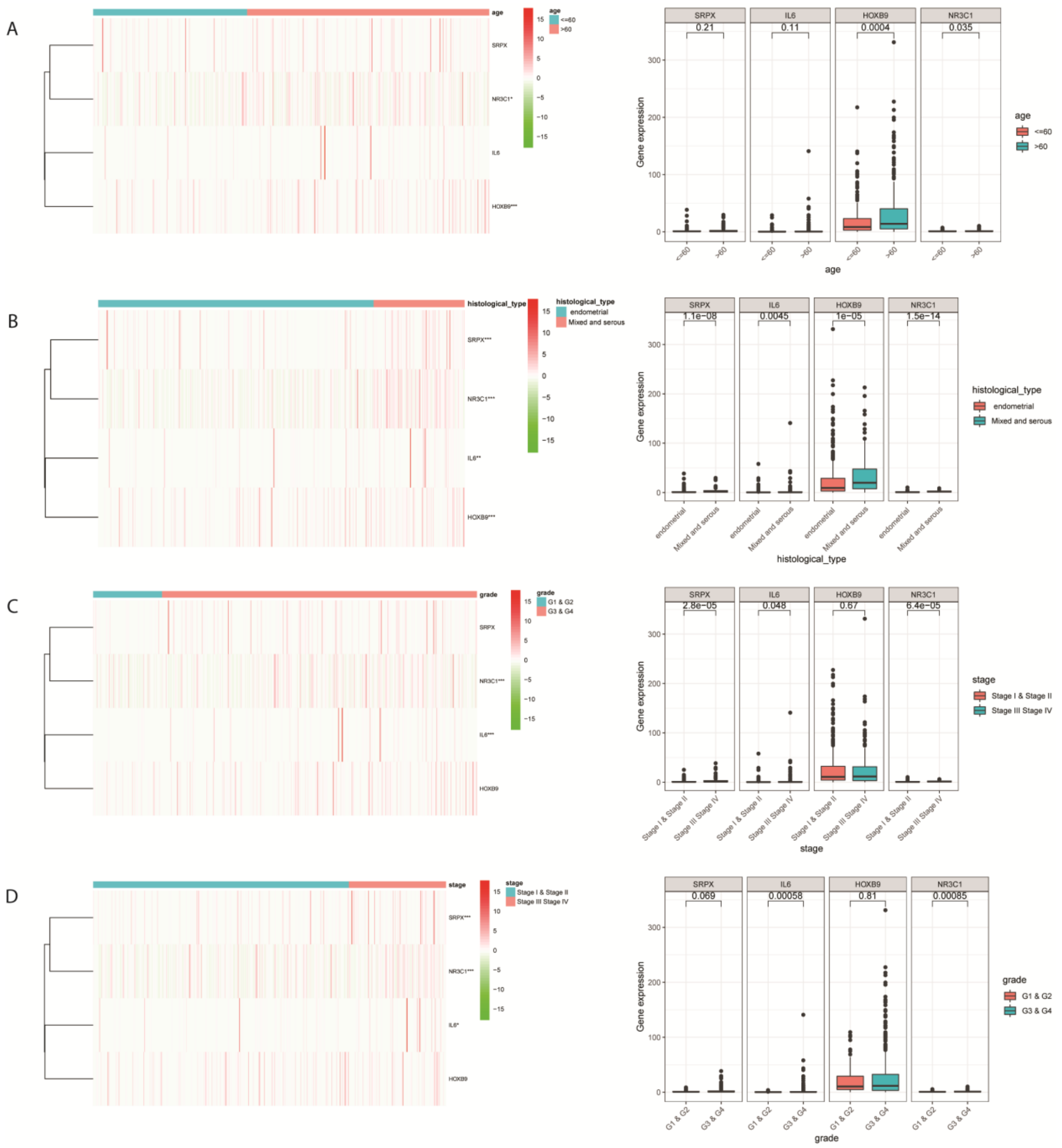

Figure S10. Heatmap and boxplots displayed expression profile of the EC hypoxia signature clustered by subdivisions of the clinical factors (age, grade, stage, and histological types) (A-D).

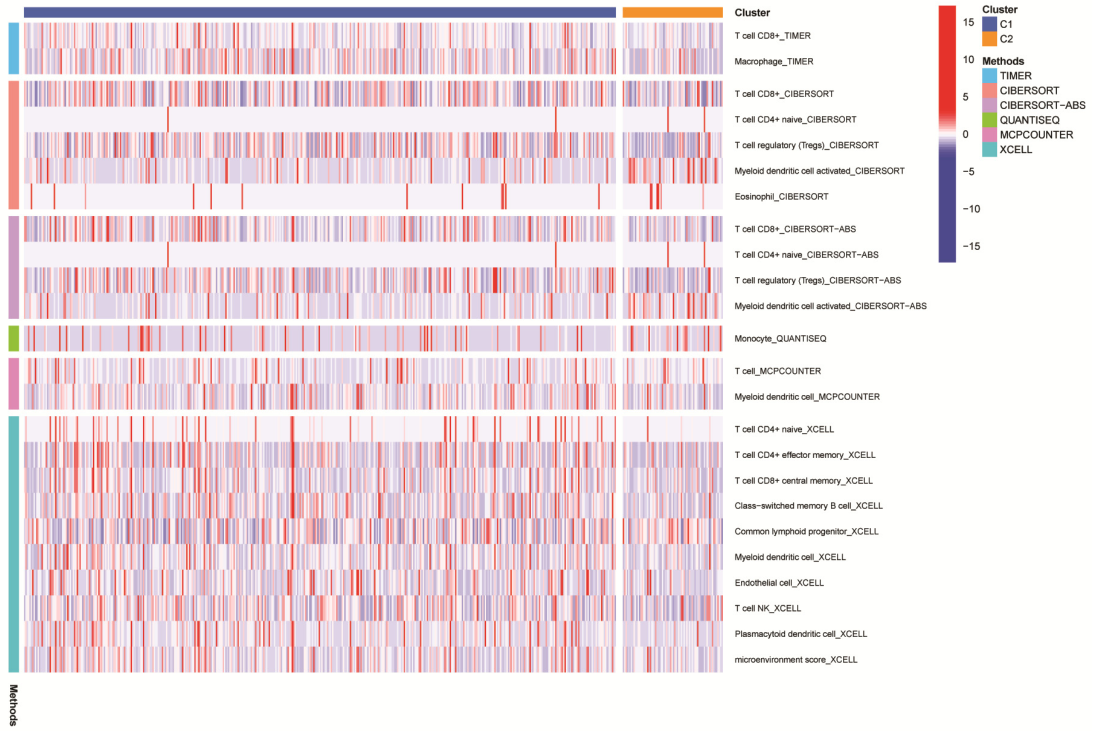

Figure S11. Methods used to compare differential immune cell expression in two molecular subtypes
